# Catalytic System for Aerobic Oxidation That Simultaneously Functions as Its Own Redox Buffer

**DOI:** 10.1021/acs.inorgchem.2c04209

**Published:** 2023-01-25

**Authors:** Xinlin Lu, Ting Cheng, Yurii V. Geletii, Craig L. Hill

**Affiliations:** Department of Chemistry, Emory University, Atlanta, Georgia30322, United States

## Abstract

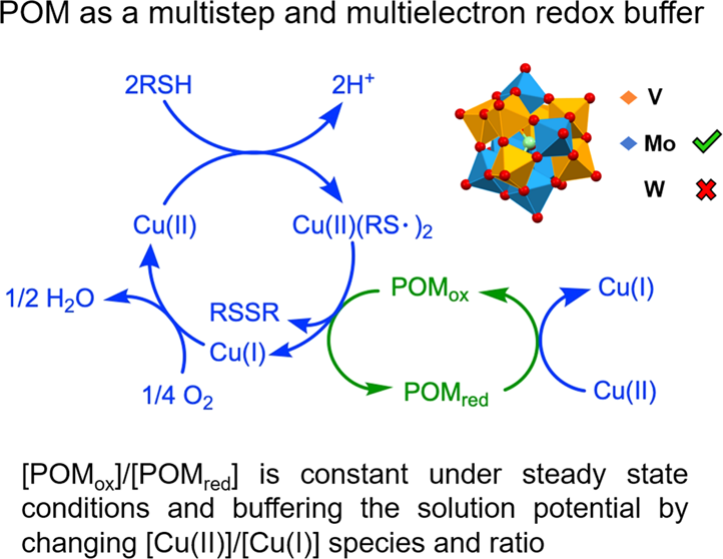

The control of the solution electrochemical potential as well as pH impacts products in redox reactions, but the former gets far less attention. Redox buffers facilitate the maintenance of potentials and have been noted in diverse cases, but they have not been a component of catalytic systems. We report a catalytic system that contains its own built-in redox buffer. Two highly synergistic components (a) the tetrabutylammonium salt of hexavanadopolymolybdate TBA_4_H_5_[PMo_6_V_6_O_40_] (**PV**_**6**_**Mo**_**6**_) and (b) Cu(ClO_4_)_2_ in acetonitrile catalyze the aerobic oxidative deodorization of thiols by conversion to the corresponding nonodorous disulfides at 23 °C (each catalyst alone is far less active). For example, the reaction of 2-mercaptoethanol with ambient air gives a turnover number (TON) = 3 × 10^2^ in less than one hour with a turnover frequency (TOF) of 6 × 10^–2^ s^–1^ with respect to **PV**_**6**_**Mo**_**6**_. Multiple electrochemical, spectroscopic, and other methods establish that (1) **PV**_**6**_**Mo**_**6**_, a multistep and multielectron redox buffering catalyst, controls the speciation and the ratio of Cu(II)/Cu(I) complexes and thus keeps the solution potential in different narrow ranges by involving multiple POM redox couples and simultaneously functions as an oxidation catalyst that receives electrons from the substrate; (2) Cu catalyzes two processes simultaneously, oxidation of the RSH by **PV**_**6**_**Mo**_**6**_ and reoxidation of reduced **PV**_**6**_**Mo**_**6**_ by O_2_; and (3) the analogous polytungstate-based system, TBA_4_H_5_[PW_6_V_6_O_40_] (**PV**_**6**_**W**_**6**_), has nearly identical cyclic voltammograms (CV) as **PV**_**6**_**Mo**_**6**_ but has almost no catalytic activity: it does not exhibit self-redox buffering.

## Abstract 2

[POM_ox_]/[POM_red_] is constant under steady-state conditions and buffers the solution potential by changing the [Cu(II)]/[Cu(I)] species and ratio.

## Introduction

Redox buffers maintain the solution electrochemical potential in a narrow range by controlling the concentration of oxidized and reduced forms of redox couples in analogy with conventional acid–base buffers that maintain the solution pH by controlling the concentration of the two forms of an acid–base couple. The fundamental equations for acid–base buffering, including but not limited to the Henderson–Hasselbach equation and redox buffering, have analogous forms.^1,2^ Redox buffering has been used to optimize performance in solid contact ion-selective electrodes,^3,4^ and functions as a key component in copper-catalyzed benzylic C–H bond functionalization.^5^ It is also a natural component in biological redox systems where the potential and more than one oxidation state of the redox-active species reversibly interconvert. Thiol-disulfide (RSH/RSSR) equilibria including the glutathione couple, GSH/GSSG, are exemplary.^6−12^ However, the formulation of a catalyst that has a built-in self-buffering capability has not been specifically reported to our knowledge.

Polyoxometalates (POMs) are attractive because they have extensive and tunable redox properties and are already successful catalysts for several commercialized organic oxidation processes. The Keggin polytungstic acids, along with PV*_n_*Mo_12–*n*_O_40_ and the binary systems, PV*_n_*Mo_12–*n*_O_40_/Pd (*n* = 1–6), are the most thoroughly studied POM catalysts.^13^ Metals other than Pd have been evaluated with polyvanadomolybdates and found to be minimally reactive (e.g., Ru, Ir) or stoichiometric (Tl). PV*_n_*Mo_12–*n*_O_40_/Pd (*n* = 1–6) has been used for the oxidation of ethylene, alkenes, and alcohols,^14,15^ and can replace corrosive chloride in the long-used Hoechst–Wacker process that uses CuCl_2_ and PdCl_2_. Our group^16^ and those of Matveev,^15^ Kozhevnikov,^13,17,18^ and Neumann^19−21^ reviewed organic substrate oxidations, including ones based on O_2_ as the terminal oxidant, catalyzed by POMs, including PV*_n_*Mo_12–*n*_O_40_/Pd systems, while Misono and Mizuno^22−24^ reviewed industrial POM-catalyzed oxidations some time ago. Thiol oxidations are also important in organic chemistry, physiological processes, and environmental science.^25−31^ Numerous catalytic and stoichiometric systems are known to selectively oxidize thiols in context with either deodorization or synthesis, including nanoparticle systems,^32,33^ POMs,^26,27,34−39^ metal–organic frameworks (MOFs),^25,40,41^ strong stoichiometric oxidants,^42−50^ and noble metals.^51−55^ Most of these systems do not use O_2_ as the terminal oxidant. In addition, most are slow, require elevated temperatures, and form side products.

The system we report here, the first POM and non-noble-metal two-component catalytic system, tetrabutylammonium salt of hexavanadopolymolybdate TBA_4_H_5_[PMo_6_V_6_O_40_] (**PV**_**6**_**Mo**_**6**_) and Cu(ClO_4_)_2_, catalyzes our target reaction in this study: the aerobic oxidative deodorization of thiols, RSH, by conversion to the desired nearly odorless disulfide, RSSR; eq 1. Cu has been chosen because it efficiently catalyzes the oxidation of reduced POMs by O_2_,^56^ and is a known catalyst of aerobic thiol oxidation.^57−64^ Remarkably, this **PV**_**6**_**Mo**_**6**_/Cu catalytic system functions as its own redox buffer, which maintains optimal conditions for the highest turnover rate.
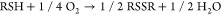
1

## Results and Discussion

We seek a catalyst for eq 1 that is fast and selective for nonodorous disulfide products and works with ambient air (O_2_) at room temperature. We used 2-mercaptoethanol as the thiol for in-depth studies because its reaction, eq 1, is facile to quantify. Table 1 summarizes the activity of Cu(ClO_4_)_2_ and multi-vanadium-substituted polytungstates and polymolybdate Keggin POMs for the oxidation of 2-mercaptoethanol (RSH) by air in acetonitrile at an ambient temperature. **PV**_**6**_**Mo**_**6**_ + Cu is the most active catalyst, achieving a turnover number (TON) of 3 × 10^2^ in 90 min. The reaction stoichiometry in eq 1 was confirmed by measurements of O_2_ and RSH consumption (see the Supporting Information (SI)) as well as the formation of the single product, bis(2-hydroxyethyl) disulfide (RSSR) (Figure S2).

**Table 1:** Air-Based Oxidation of 30 mM 2-Mercaptoethanol Catalyzed by Different POM Systems^a^

catalyst^e^	Cu concentration^a^ (mM)	conversion^b^ (%)	TON × 10^–2 ^c	TOF × 10^3^ (s^–1^)^d^
Cu(ClO_4_)_2_	0.5	9	0.03	0.6
**PV**_**3**_**W**_**9**_	0.5	13	0.42	7.8
**PV**_**6**_**W**_**6**_	0.5	16	0.57	11
**V**_**10**_**O**_**28**_	0.5	12	0.36	6.7
**PMo**_**12**_	0.5	9	0.03	0.6
**PV**_**6**_**Mo**_**6**_	0.5	90	2.9	53
**PVMo**_**11**_	0.8	10	0.30	5.6
**PV**_**2**_**Mo**_**10**_	0.8	12	0.36	6.7
**PV**_**3**_**Mo**_**9**_	0.8	16	0.48	8.9
**PV**_**4**_**Mo**_**8**_	0.8	86	2.6	48
**PV**_**6**_**Mo**_**6**_	0.8	100	3.0	56
**PV**_**6**_**Mo**_**6**_^f^	0.5	60	3.0	55

a Conditions: POM (0.1 mM), 2-mercaptoethanol (30 mM), and acetonitrile (5 mL) at room temperature under air.

b Conversion was measured after 90 min.

c Turnover number (TON = moles of 2-mercaptoethanol consumed per mol of POM) was measured after 90 min.

d Turnover frequency, TOF = TON/(reaction time).

e For the POM chemical formulas, see the POM synthesis section in the SI.

f 50 mM 2-mercaptoethanol.

Based on the results in Table 1, the catalytic activity of molybdates increases as the number of vanadium atoms in the POM increases. The same trend was reported by Kozhevnikov et al.^65^ for the oxidation of 2,3,6-trimethylphenol (TMP) by O_2_ in AcOH–H_2_O catalyzed by H_3+*n*_PMo_12–*n*_V*_n_*O_40_. They assumed that the active species is vanadate, VO_2_^+^, and its dissociation from the parent POM, PMo_12–*n*_V*_n_*O_40_^(3+*n*)–^, is more pronounced with increasing numbers of vanadium atoms.^66^ If free vanadate is the active center, the catalytic activity of POM should be similar to that of the same concentration of vanadate.^65^ Therefore, we compared the activity of sodium metavanadate alone (NaVO_3_) and the mixture of NaVO_3_ with **PV**_**6**_**Mo**_**6**_ (Figure S3). NaVO_3_ alone was catalytically inactive, and there was no difference in activity between POM with and without NaVO_3_. These two arguments strongly suggest that free monomeric vanadium species are not involved in catalysis under these conditions. Further ^31^P NMR of a mixture of **PV**_**6**_**Mo**_**6**_ and **PVMo**_**11**_ shows only stack peaks of these two POMs but not a redistribution of isomers. Aging **PV**_**6**_**Mo**_**6**_ overnight does not show additional NMR peaks (Figure S22). These results suggest that **PV**_**6**_**Mo**_**6**_ is thermodynamically stable in acetonitrile over the reaction timescale. Reduction generates paramagnetic V(IV), which cannot be observed by NMR. However, we were able to observe the fairly constant reduced states of the POM under turnover conditions by UV–vis spectroscopy (vide infra). V(IV) has a totally different UV–vis absorption band than the V(IV)-to-Mo(VI) intervalence charge-transfer (IVCT) band in the Keggin POM. If free vanadium outside the POM is dominant during the catalytic reaction, it is impossible for us to observe the fairly constant reduction states based on the V(IV)-to-Mo(VI) IVCT band. Therefore, we make an assumption here that vanadium outside the POM is not significant in this system. The difference in our multicomponent catalyst system here and the classic studies of Kozhevnikov in aqueous media is not surprising because the solvation energy of VO_2_^+^ in acetonitrile is much lower than in water, and thus VO_2_^+^ dissociation from the polyanion in CH_3_CN is much less favorable. This is consistent with Kozhevnikov’s results—changing the solvent to acetonitrile shuts down the catalytic activity.^65^

**Figure 1:**
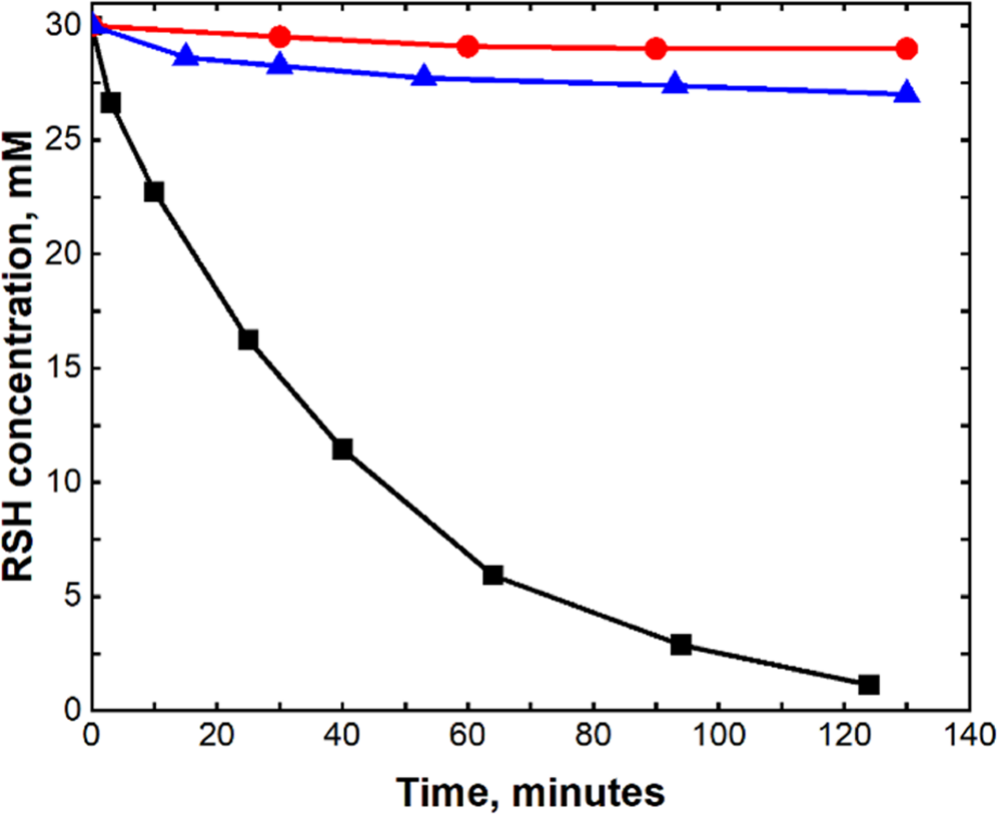
Kinetics of 30 mM 2-mercaptoethanol oxidation by air (1 atm, 24 °C) in acetonitrile catalyzed by 0.1 mM **PV**_**6**_**Mo**_**6**_ (red), by 0.5 mM Cu(ClO_4_)_2_ (blue), and by a combination of 0.1 mM **PV**_**6**_**Mo**_**6**_ + 0.5 mM Cu(ClO_4_)_2_ (black).

One of the most interesting findings is the dramatic synergism of the catalytic activity of Cu(ClO_4_)_2_ and PMo_12–*n*_V*_n_*O_40_^(3+*n*)–^ (*n* = 1–4, 6)—the activity of the mixture of two catalysts is much higher than a sum of their individual activities (Figure 1). To explain this phenomenon, we have studied the function of POM and Cu by kinetics and electrochemical methods and probed the processes constituting the reaction mechanism. The system with the highest catalytic activity, Cu/**PV**_**6**_**Mo**_**6**_, was chosen for these studies.

### Features of the Catalytic Aerobic Oxidation of 2-Mercaptoethanol

**Figure 2:**
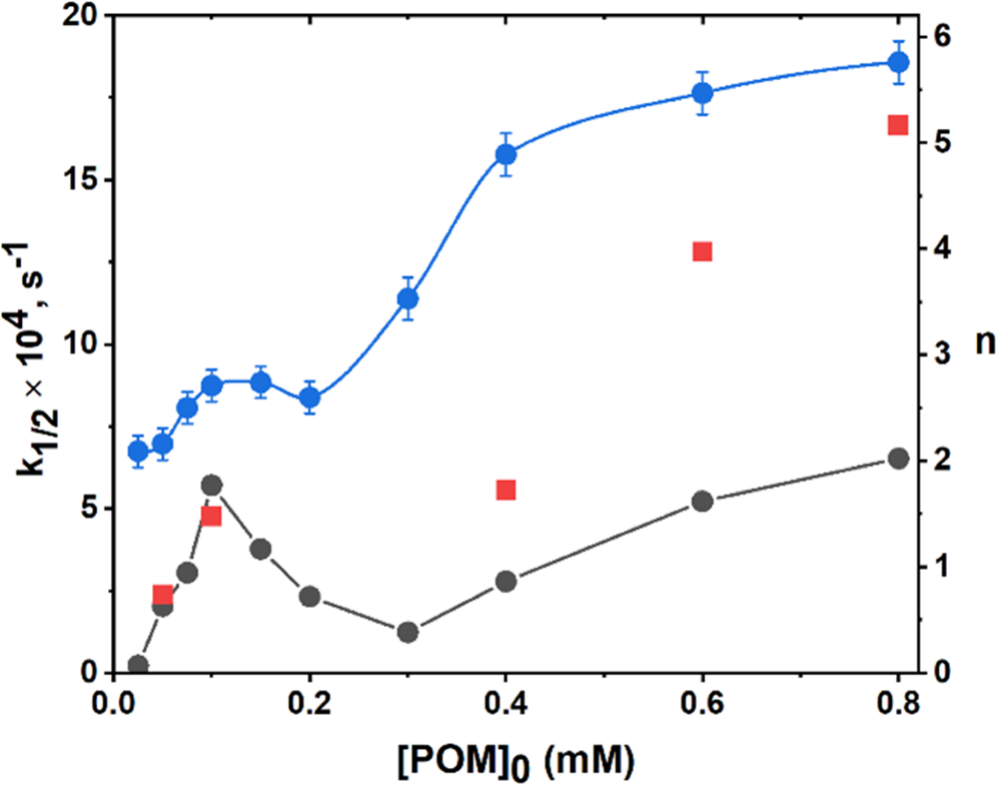
Dependence of *k*_1/2_ and *n* as a function of initial POM concentration. Conditions: 0.5 mM Cu(ClO_4_)_2_ and 30 mM RSH under air (black) and O_2_ (red); the reduction state of **PV**_**6**_**Mo**_**6**_ is expressed in *n* (blue).

Figure S4a shows that there is negligible inhibition of the rate by the accumulating product, the disulfide RSSR. Moreover, we found no significant effect of either water or ionic strength on the rates (Figure S4b,c). Therefore, no extra water nor other salts were added in our experiments. The kinetics of 2-mercaptoethanol oxidation depends on concentrations of RSH, POM, Cu, and O_2_, which makes it extremely hard to quantitively analyze. Moreover, we found an unusual dependence on POM initial concentration; Figure 2. We measured the time required to reach 50% conversion of RSH, *t*_1/2_ = 1/*k*_1/2_, and used *k*_1/2_ to quantify the reaction rate (see the SI for more details; Figure S5). The activity increases with [POM], in the range of 0–0.1 mM and at 0.3–0.8 mM [**PV**_**6**_**Mo**_**6**_]_0_. In the middle concentration range of 0.1–0.3 mM, *k*_1/2_ shows a negative dependence on [**PV**_**6**_**Mo**_**6**_]_0_. The replacement of air with pure O_2_ does not change the rate at low [**PV**_**6**_**Mo**_**6**_]_0_ <0.1 mM but increases the rate at higher concentrations (Figure 2, red). At 0.8 mM [**PV**_**6**_**Mo**_**6**_]_0_ and 0.5 mM Cu(ClO_4_)_2_, the rate is 2.5-fold higher under air. This kinetic behavior was hard to understand until we managed to determine the reduction state, *n*, of the POM under turnover conditions. The change in POM concentration changes not only the quantity of the catalyst in the solution but also the distribution of POM reduction states and thus the solution potential. These facts, in turn, explain the unusual kinetic dependences. The reduction state curve (blue) and *k*_1/2_ exhibit a similar trend, suggesting that they correlate with each other. The measurement of the POM apparent reduction state, *n*, and the relationship between *n* and *k*_1/2_ are described below.

### Electrochemistry

**Figure 3:**
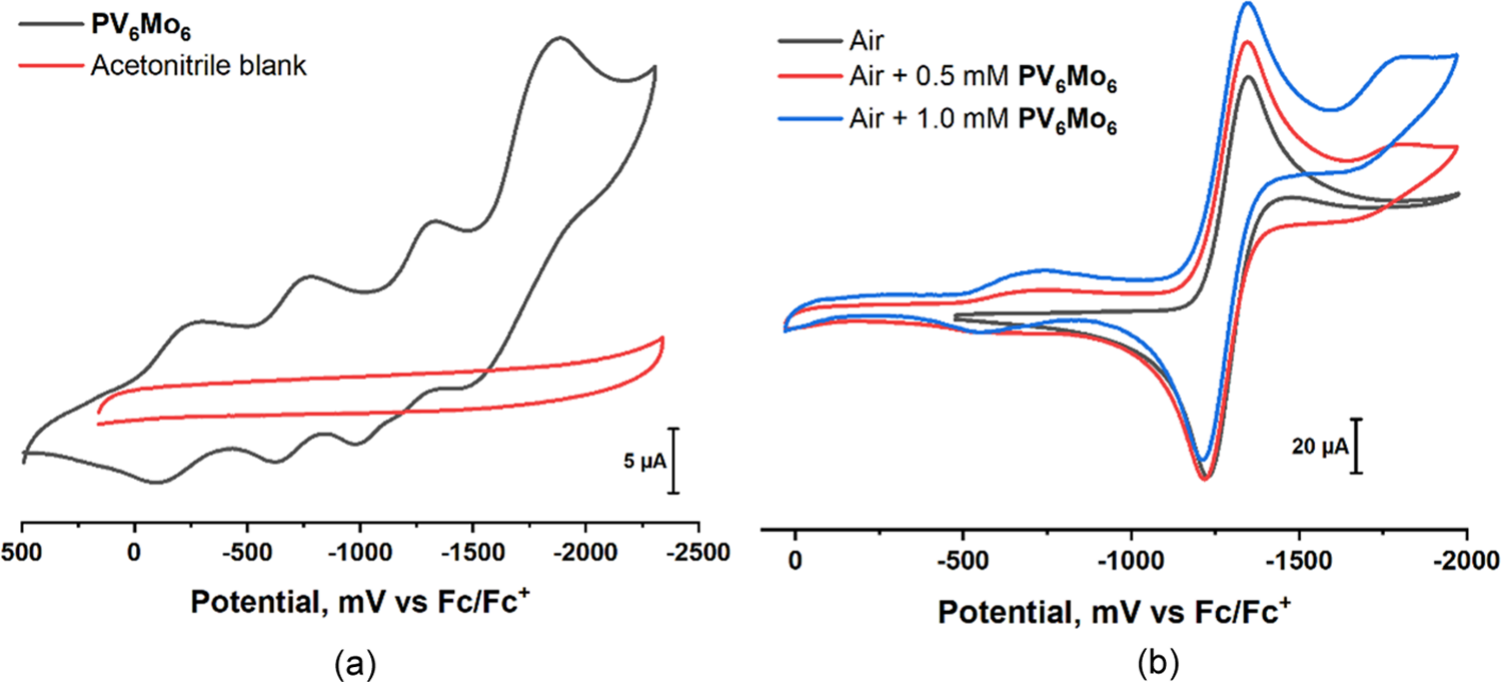
(a) Black: cyclic voltammograms (CV) of 0.5 mM **PV**_**6**_**Mo**_**6**_ on a glassy carbon electrode in acetonitrile under argon. Red: CV of acetonitrile blank. (b) CV of air-saturated acetonitrile. Black: 0 mM **PV**_**6**_**Mo**_**6**_; red: 0.5 mM **PV**_**6**_**Mo**_**6**_; blue: 1.0 mM **PV**_**6**_**Mo**_**6**_. Conditions: 100 mM *n*-Bu_4_NPF_6_, scan rate = 100 mV s^–1^, and *T* = 298 K.

Electrochemical studies reveal some reaction thermodynamics. The cyclic voltammograms (CVs) of **PV**_**6**_**Mo**_**6**_, Figure 3a, show three reversible peaks of the same height and a fourth peak that is high, broad, and quasi-reversible. The differences between two successive *E*_1/2_^0^ are about 500 mV. For comparison, these peak-to-peak separations in water are 180–220 mV and, in general, depend on the dielectric constant of the solvent (37 and 81 for acetonitrile and water, respectively).^67^

The CV of an air-saturated solution shows a reversible peak overlapping with the third peak of POMs (Figure 3b). The CV of the air-saturated solution taken in the presence of 1.0 mM **PV**_**6**_**Mo**_**6**_ is the same as in the absence of the POM and is assigned to the one-electron-reduction product of O_2_, superoxide. The ratio of cathodic to anodic currents of these peaks is close to 1. The reversibility of this peak indicates that the reaction product, O_2_^•–^, is relatively stable under these conditions and that the addition of protonated **PV**_**6**_**Mo**_**6**_ does not affect the lifetime of O_2_^•–^ (the ratio of the cathodic to the anodic current remains equal to 1). Based on these data, we can conclude that the protonated POM, in acetonitrile, unlike water, is not strong enough to protonate superoxide. It is well known in aqueous media that protonated superoxide, HO_2_^•^, rapidly forms dioxygen and hydrogen peroxide.^68^ These findings are consistent with the fact that H^+^ in protonated **PV**_**6**_**Mo**_**6**_ in acetonitrile does not dissociate and thus the POM is not a strong acid under these conditions.

**Figure 4:**
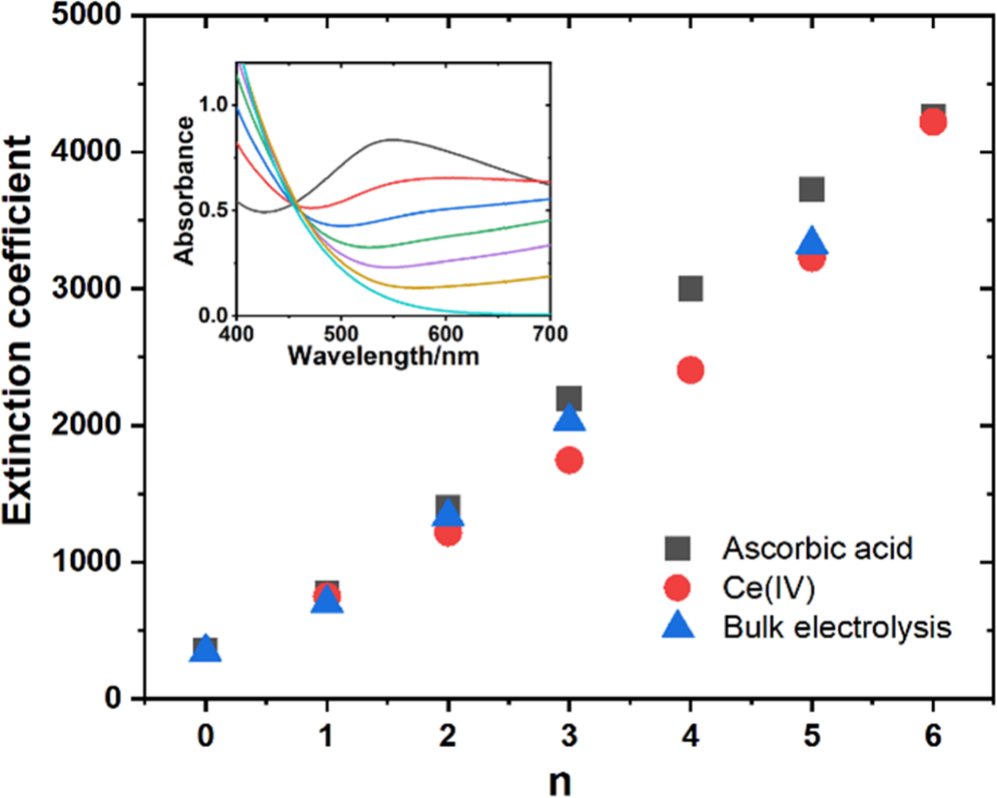
Dependence of the apparent extinction coefficient of **PV**_**6**_**Mo**_**6**_ at 550 nm on the number of electrons, *n*, transferred from ascorbic acid (black squares), after reoxidation by cerium ammonium nitrate (CAN) (red circles) and after bulk electrolysis (blue triangles). Inset: UV–vis spectrum of ascorbic acid titration. The size of the icons is equal to the error bars.

To confirm the number of electrons involved in the redox reactions, we performed bulk electrolysis of **PV**_**6**_**Mo**_**6**_ at controlled potentials (Figure S6 and Table S1). The chronocoulometric results show that all three peaks for **PV**_**6**_**Mo**_**6**_ are one-electron. The electrolysis at −2000 mV does not have an identifiable endpoint and therefore is not informative. The spectra of solutions after electrolysis are the same as those after titration by ascorbic acid (Figure 4). In addition, rotating disk electrode (RDE) voltammetry was used to determine the number of electrons in the fourth peak (Figure S7). The *E*_1/2_ of the four processes in RDE is consistent with CV data (Table S2) and independent of the rotation rate. The Levich plot (*i*_L_ versus ω^1/2^) for all four processes is linear, indicating mass transport control. The relative ratio of limiting currents is I/II/III/IV = 1:1.1:1.1:4 ± 0.5.

### Measurements of the **PV_6_Mo_6_** Reduction State under Turnover Conditions

The apparent reduction state of **PV**_**6**_**Mo**_**6**_ during the reaction can be defined as the average number of electrons, *n*, transferred to the POM (eqs S5 and S6 in the SI). To measure the **PV**_**6**_**Mo**_**6**_ apparent reduction state in the course of the reaction, the UV–vis absorbance at 550 nm was monitored as a function of time. Figure S8a shows examples of the POM at a high reduction state (*n* = 5.9), remaining almost constant (steady-state) to about 75% conversion of RSH, then slowly decreasing. Figure S8b is an example of **PV**_**6**_**Mo**_**6**_ remaining almost constant in the low reduction state (*n* = 2.2) until reaction completion. The initial reduction process is very fast and has been studied separately (see below). Thus, we have focused on steady reduction states for all measurements under turnover conditions. To establish the correlation between *n* and absorbance, we titrated fully oxidized **PV**_**6**_**Mo**_**6**_ with ascorbic acid (Figure 4). In water, the removal of the first electron from ascorbic acid is a reversible pH-dependent process producing the ascorbate radical. However, oxidation of ascorbate radical includes irreversible nonredox transformation.^69,70^ The irreversibility of this reaction is the key property of ascorbic acid, making it a very strong two-electron stoichiometric reducing agent.

Spectral changes and the formally calculated extinction coefficient at 550 nm in the process of titrating **PV**_**6**_**Mo**_**6**_ with freshly prepared ascorbic acid are shown in Figure 4. The spectra of POM under turnover conditions are given in Figure S20, which are identical to the titration spectra. Subsequently, we titrated the fully reduced POM with the strong one-electron oxidant Ce(IV) (cerium ammonium nitrate or “CAN”). The titration curves of the reduced POMs (Figure 4) reproduce the lines for reductions by ascorbic acid and confirm that ascorbic acid functions in these acetonitrile solutions as a two-electron reductant as in water. Thus, **PV**_**6**_**Mo**_**6**_ can be reversibly reduced by six electrons under these conditions.

Finally, the number of electrons, *n*, transferred to POM in the range 1–5 is linearly dependent on absorbance at 550 nm normalized by the **PV**_**6**_**Mo**_**6**_ concentration (apparent extinction coefficient). This calibration has been used to convert the absorbance to *n* (Figure S9a). The UV–vis spectra from 400–1100 nm are given in Figure S9b. A broad band centered around 1000 nm gradually increases and reaches a plateau from *n* = 1 to 5 and decreases at *n* = 6. In addition, another band increases linearly from *n* = 1 to 6. To assign the bands, we start from the UV–vis spectra of reduced **PVMo**_**11**_ in acetonitrile. Bulk electrolysis titration of **PVMo**_**11**_ in acetonitrile is given in Figure S21. The one-electron-reduced **PVMo**_**11**_ has a broad absorbance centered around 650 nm that can be assigned as the V^IV^-to-Mo^VI^ intervalence charge-transfer (IVCT) band.^71,72^ The two-electron-reduced **PVMo**_**11**_ has a high-extinction-coefficient absorbance centered around 700 nm, which can be assigned to the Mo^VI^-to-Mo^V^ IVCT band.^73,74^ Further, the one- and two-electron-reduced forms of **PV**_**2**_**Mo**_**10**_ exhibit a broad absorption centered around 600 that can be assigned as a V^IV^-to-Mo^VI^ IVCT band and a shoulder absorption centered around 850 nm that can be assigned as a V(IV) d–d transition.^72^ Compared with **PVMo**_**11**_ and **PV**_**2**_**Mo**_**10**_, the broad band around 1000 nm for the one- to four-electron-reduced forms of **PV**_**6**_**Mo**_**6**_ where *n* = 1–4 is tentatively assigned as a V^IV^-to-V^V^ IVCT band.^75^ One strong argument for this assignment is that this absorption band disappears when *n* = 6, where all V(V) atoms have been reduced to V(IV). In addition, V(IV) d–d transition should also have a contribution around 850 nm. The absorption around 550–600 nm that linearly increases can thus be assigned as a V^IV^-to-Mo^VI^ IVCT band. No high-extinction-coefficient Mo^VI^-to-Mo^V^ IVCT band at 700 nm is observed when *n* = 6, suggesting that all six electrons result in the reduction of V^V^ to V^IV^.

### Redox Buffering by **PV_6_Mo_6_**

**Figure 5:**
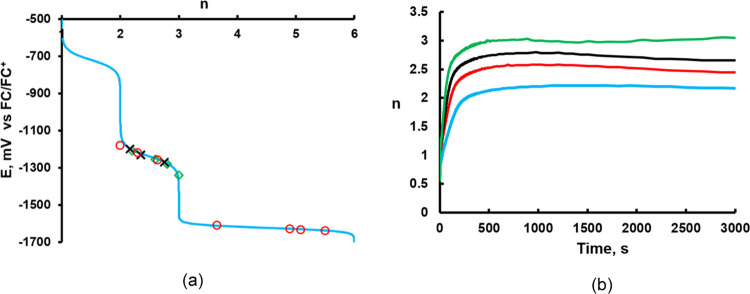
(a) Theoretical values of chemical solution potentials as a function of the reduction state of **PV**_**6**_**Mo**_**6**_ (blue line), and the experimental values for (i) 0.1 mM **PV**_**6**_**Mo**_**6**_, 0.5 mM Cu(ClO_4_)_2_, and different [RSH]_0_ (green diamonds); (ii) 0.1 mM **PV**_**6**_**Mo**_**6**_, 30 mM RSH, and different Cu(ClO_4_)_2_ (data from Figure S5; black crosses); (iii) 0.5 mM Cu(ClO_4_)_2_, 30 mM RSH, and different concentrations of **PV**_**6**_**Mo**_**6**_ (data from Figure 2; red circles). (b) Change of the apparent reduction state of **PV**_**6**_**Mo**_**6**_ as a function of time during thiol oxidation by air with 0.1 mM **PV**_**6**_**Mo**_**6**_ and 0.5 mM Cu(ClO_4_)_2_, [RSH] = 20 (blue), 30 (red), 40 (black), and 50 mM (green).

The apparent reduction state of **PV**_**6**_**Mo**_**6**_, *n*, under turnover conditions depends on the initial concentrations of POM, RSH, and Cu(II) (Figures 2, 5b, and S5c) but remains almost constant under steady-state conditions. The dependence of *n* on the chemical solution potential *E* is shown in Figure 5a (see the SI for details; Figure S10). All of the measured reduction states under different conditions have been placed on the curve, revealing that all of these values fall into two potential ranges: *E* = −(1180–1340) and −(1600–1680) mV (Figure 5a). Thus, a correlation is evident between the thermodynamic parameter, *n*, and the reaction rate, *k*_1/2_, in Figure 2. The first positive [POM]_0_ dependence range 0–0.1 mM is when *E* = −(1180–1340) mV and the second positive [POM]_0_ dependence range 0.3–0.8 mM is when *E* = −(1600–1680) mV. The middle negative dependence on [POM]_0_ is the transition between them. **PV**_**6**_**Mo**_**6**_ resides in different reduction states corresponding to different solution electrochemical potential ranges. *k*_1/2_ has two distinct positive [POM]_0_ dependence ranges indicating different rate-limiting steps during the overall reaction induced by solution potential changes. The rate-limiting step may involve reoxidation of Cu(I) by O_2_ as we observe different [O_2_] dependences in the two ranges (Figure 2). The low POM concentration range (0–0.1 mM) does not respond to [O_2_] change, suggesting that the rate-limiting step in this range does not involve reoxidation by O_2_. However, the reaction rate under pure O_2_ of the high POM concentration range (0.3–0.8 mM) is 2.5-fold higher than under air, indicating that the rate-limiting step involves O_2_ reactions. Since under O_2_, the reaction is not 5 times faster than under air; there is no conventional single well-defined rate-limiting step in this concentration regime.

The change of Gibbs free energy (expressed by the electrochemical solution potential, Δ*E* in mV) in the course of the reaction of thiol oxidation, eq 1, is
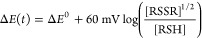
2
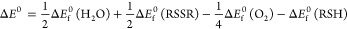
3where Δ*E*_f_^0^ are the standard Gibbs energies of formation.

The electrochemical solution potential in the catalytic reaction is in eq 4.
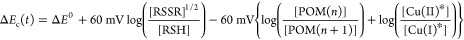
4Since the ratio [POM(*n*)]/[POM(*n* + 1)] remains constant under steady-state conditions, the contribution to Δ*E*_c_(*t*) of the [Cu(II)*]/[Cu(I)*] redox couple becomes important. [Cu(II)*] and [Cu(I)*] are the total concentrations of all Cu(II) and Cu(I) complexes present.

### Thermodynamics, Speciation, and Catalytic Cycle of Copper Complexes

Copper speciation in acetonitrile has been studied previously. Copper(I) is strongly complexed by acetonitrile, and the stable salt [Cu(NCCH_3_)_4_](ClO_4_) has been prepared.^76^ Copper(II) exists in acetonitrile as a hexacoordinated distorted octahedron.^77^ Both Cu(II) and Cu(I) can bind RSH and form polymeric structures.^30,76^ When the acetonitrile solution contains water, Cu(II) should bind two RSH per ion and Cu(I) should bind an average of one RSH per ion;^30,76^eqs 5, 6, and 8. That Cu(II) can bind 2 equiv of RSH in acetonitrile is proved using cyclic voltammetry and titration methods (Figures S11 and S19). The formation of copper complexes with RSH results in the shift of the apparent potential to negative values (see Figure S11 for more details). Based on our data, we suggest the following reaction mechanism
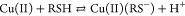
5
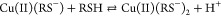
6
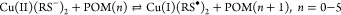
7
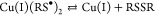
8
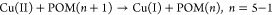
9
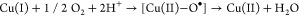
10All reactions, except in eqs 9 and 10, are reversible. Two important experiments are required before discussing the mechanism. The overall reaction mechanism includes the POM(*n*)/POM(*n* + 1) distribution and the multistep reaction of O_2_ with Cu(I), which, collectively, are too complicated to analyze. Thus, we separate the overall process into the reaction under Ar with RSH (POM(*n*) → POM(*n* + 1)) and the reaction with O_2_ but no RSH (POM(*n* + 1) → POM(*n*)) to understand the function of copper.

### Cu(II) Catalysis of **PV_6_Mo_6_** Reduction by RSH under Ar

**Figure 6:**
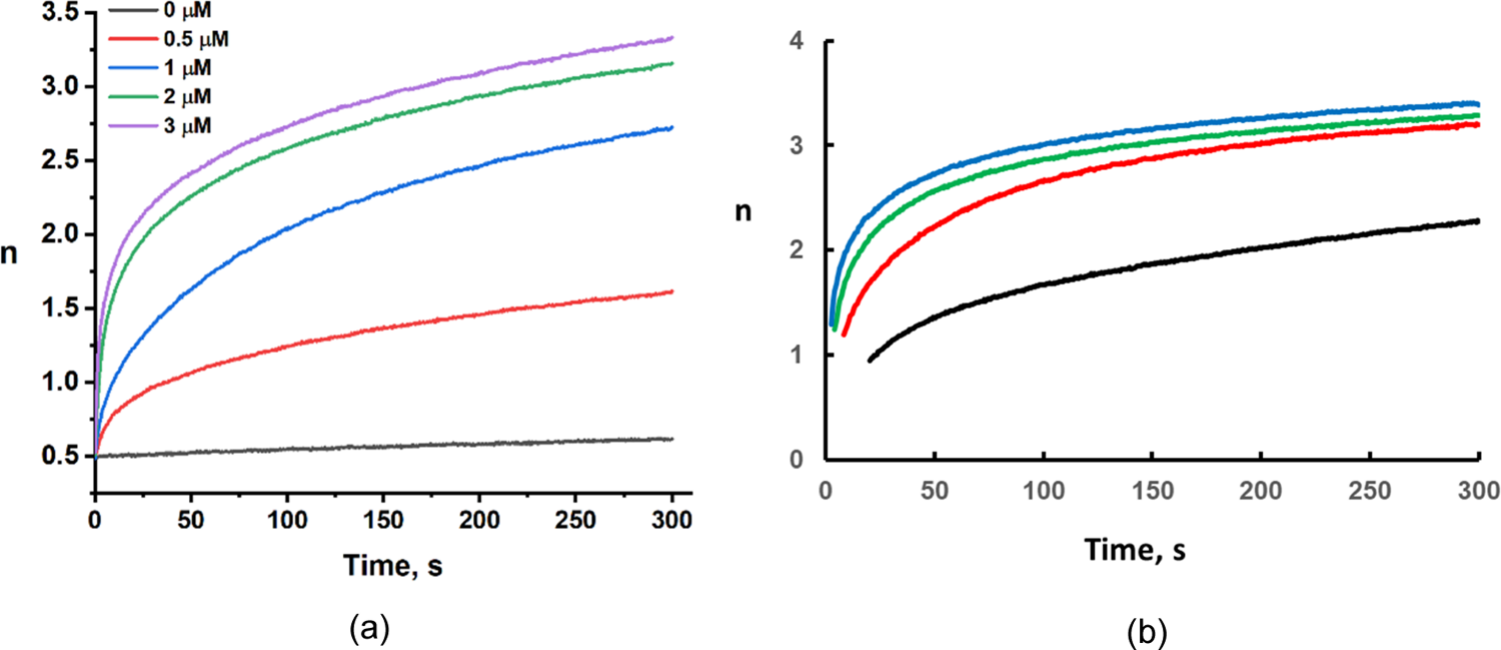
(a) Effect of Cu(II) concentration on **PV**_**6**_**Mo**_**6**_ reduction by 2-mercaptoethanol in acetonitrile followed at 550 nm (expressed in the number of electrons acquired, *n*). Conditions: **PV**_**6**_**Mo**_**6**_ (0.5 mM) and 2-mercaptoethanol (25 mM) under argon. (b) Kinetics of 0.5 mM **PV**_**6**_**Mo**_**6**_ reduction (expressed in the number of electrons acquired, *n*) by 12.5 (black), 25 (red), 37.5 (green), and 50 mM (blue) of RSH in the presence of 1.0 μM Cu(ClO_4_)_2_ under Ar.

The data in Figure 5, which reflects turnover conditions, show that the reduction state of **PV**_**6**_**Mo**_**6**_, *n*, quickly increases with time and reaches the plateau within 5 min. To study this initial process, we excluded reoxidation by replacing air with Ar and looked at the kinetics of changing *n* by following the absorbance at 550 nm. This reaction appeared to be extremely efficiently catalyzed by micromolar amounts of Cu(ClO_4_)_2_; Figure 6a. During the initial fast step, **PV**_**6**_**Mo**_**6**_ is reduced by two to three electrons by 25–50 mM RSH (Figure 6b), which is consistent with the mechanism in eqs 5–8. Without O_2_, the regeneration of Cu(II) is very likely eqs 11 and 12.^49^
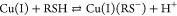
11
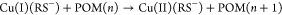
12Earlier, we found that submicromolar amounts of Cu(II) are always present in water.^56,78,79^ Here, we use the same method to prove that no RSH oxidation takes place with the POM alone without Cu(II). Neocuproine, 2,9-dimethyl-1,10-phenanthroline (DMP), was employed to chelate the trace amount of Cu(II); around 10 μM DMP completely stopped the reaction (Figure S12). This further confirms that the Cu(II)/RSH complex and not RSH is the reducing species, and the entire reduction process under turnover conditions can be described by eqs 5–8.

### Reaction of Reduced **PV_6_Mo_6_** with O_2_ Catalyzed by Cu(II)

**Figure 7:**
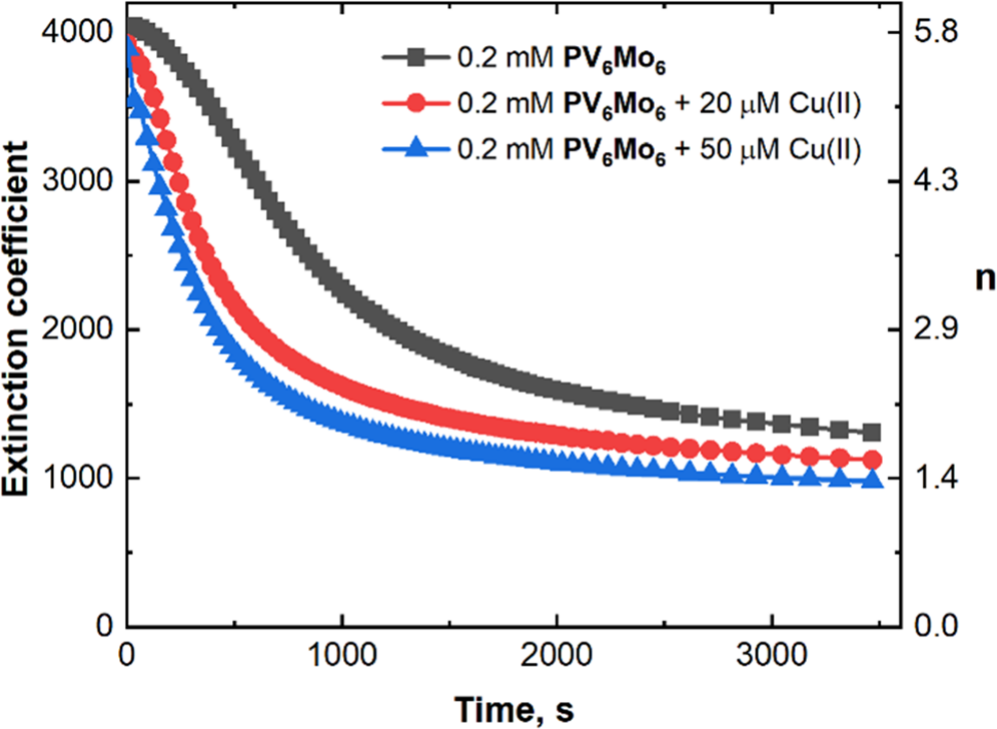
Effect of Cu(II) concentration on reduced **PV**_**6**_**Mo**_**6**_ (0.2 mM solution) by O_2_-based oxidation, followed by the decrease of absorbance at 550 nm. The six-electron reduced **PV**_**6**_**Mo**_**6**_ was obtained by adding 3 equiv of ascorbic acid (six electrons transferred).

In previous work, we found that reoxidation of one-electron reduced POMs, α-AlW_12_O_40_^6–^, α-SiW_12_O_40_^5–^, and α-PW_12_O_40_^4–^, is catalyzed by copper.^56,78−80^ In this study, **PV**_**6**_**Mo**_**6**_ was reduced (six electrons) with 3 equiv of ascorbic acid under Ar. Subsequently, the reduced **PV**_**6**_**Mo**_**6**_ was reoxidized by purging O_2_ in the presence of different concentrations of Cu(ClO_4_)_2_. We found that the oxidation of the reduced **PV**_**6**_**Mo**_**6**_ by O_2_ is also catalyzed by small amounts of copper in the absence of RSH (Figure 7). The short induction period is caused by the presence of a small amount of unreacted ascorbic acid. In contrast to the previous study in aqueous conditions,^10^ Cu(II) in acetonitrile is a strong oxidant relative to reduced **PV**_**6**_**Mo**_**6**_. The standard reduction potential *E*_0_ of the Cu(II)/Cu(I) couple measured by CV is unusually high, 675 mV (Figure S11), which is consistent with the literature value 950 mV versus the saturated calomel electrode (SCE).^81^ Although reduced POM alone can react with O_2_, this process is far slower when the catalyst, copper, is also present. Therefore, the copper-catalyzed POM reoxidation process can be described by eqs 9 and 10 (see the discussion below for the reaction of Cu(I) and O_2_). This result and the CV study (Figure S13) prove that Cu(I) can react with O_2_ in acetonitrile to regenerate Cu(II). Thus, Cu(II) has a remarkable ability to catalyze two distinct processes, the reduction of POMs by thiol and the reoxidation of the resulting reduced POMs by O_2_.

The standard reduction potential of the Cu(II)/Cu(I) couple, *E*_0_, is very high in acetonitrile, 675 mV versus Fc^+^/Fc, and the direct reaction of Cu(I) with O_2_ to form superoxide is thus thermodynamically very unfavorable. The oxidation of Cu(I) likely proceeds through the formation of an intermediate Cu(I)···O_2_ complex, which reacts with a second Cu(I) to form an unstable di-copper peroxo intermediate. The homolysis of the O–O bond results in the formation of two formally Cu(III) intermediates, which we write as [Cu(II)–O^•^]. Recent multiedge X-ray absorption spectroscopic (XAS) and density functional theory (DFT) studies put into question the existence of the Cu(III) species in most coordination environments.^82^ This oxidized Cu center would oxidize the reduced POMs by one electron and generate Cu(II).^83^ Cu(II), as a strong one-electron oxidant, oxidizes a second reduced POM, regenerating Cu(I).

Additional electrochemical measurements provide further evidence for the general processes of the mechanism. Figure S13 shows the CVs of Cu under Ar and O_2_ at different scan rates. The anodic and cathodic current ratio *i*_a_/*i*_c_ of the Cu(II)/Cu(I) peak decreases with the decreasing scan rate. The anodic current approaches 0 at 20 mV s^–1^. This indicates that Cu(I) will be consumed by O_2_ when the reaction time increases. Furthermore, the symmetric anodic peak belonging to the absorbed Cu(I)/Cu(0) peak disappears when O_2_ is present.^84,85^ These results strongly suggest that Cu(I) forms a complex with O_2_. This mechanism does not require the presence of RSH in the solution, which is in agreement with the experimental data in Figure 7.

### Overall Mechanism under Turnover Conditions

**Scheme 1:**
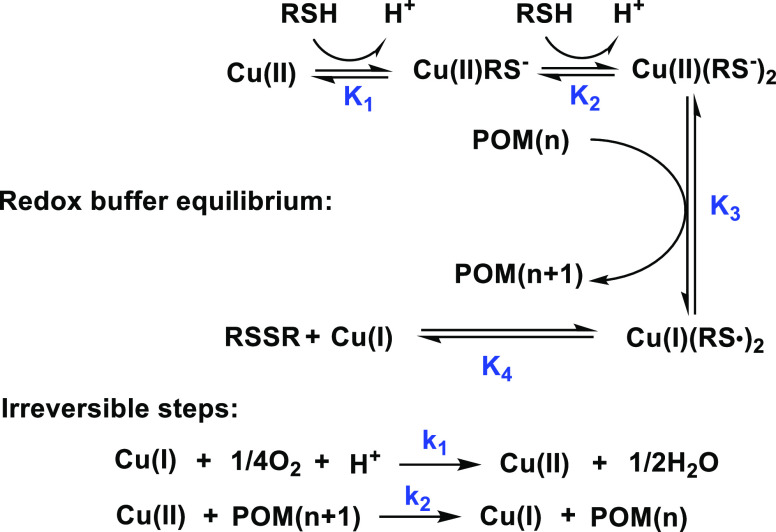
Catalytic Cycle of the Cu(II)/Cu(I) Couple

Given the current level of knowledge of POM speciation in the presence of Cu and also the substrate (RSH), it is not feasible to construct a quantitative mechanism scheme incorporating all active species during catalytic turnover (POM, Cu, RSH, O_2_). However, Scheme 1 shows the key pathways consistent with all our data in this study and with the equations above.

Most of the experiments were conducted with a RSH/Cu concentration ratio of 60, and we proved that RSH alone could not reduce POM. Therefore, we considered Cu(II)(RS^–^)_2_ as the only active reducing agent for simplicity.^54,55,61^ We assume that the dissociation of the thiolate complex Cu(II)(RS^–^) into Cu(I) and RS^•^ is unfavorable and that reaction 8 driven by S–S bond formation is dominant. The regeneration of Cu(II) occurs either by the reaction of Cu(I) with O_2_, eq 10, or by the reaction of Cu(I)(RS^–^) with POM(*n*), eqs 11 and 12. We should note that the reaction under Ar catalyzed by micromolar Cu is different from the overall reaction under turnover conditions catalyzed by millimolar Cu. The overall reaction under turnover conditions consumes O_2_ (Figure S1) and has trace conversion under Ar. Therefore, in aerobic turnover conditions, eq 10 is the dominant pathway to regenerate Cu(II); eqs 11 and 12 can be neglected.

The full analysis of the catalytic cycle is very complicated. For simplicity, we assume that all reactions between copper, POM, and RSH/RSSR are in thermodynamic equilibrium, except the irreversible reactions of Cu(I) with O_2_ and Cu(II) with the reduced forms of POM.

The presence of a catalyst does not change the overall reaction thermodynamics. Since [POM(*n*)]/[POM(*n* + 1)] remains constant under steady-state conditions, the speciation and ratio of Cu(II)/Cu(I) should change. The ratio of oxidized-to-reduced POM, [POM(*n*)]/[POM(*n* + 1)], controls the distribution of the Cu complexes. This is an internal redox buffer. This is similar to a conventional buffer where the ratio of protonated-to-deprotonated forms controls the pH. Since **PV**_**6**_**Mo**_**6**_ can keep solution electrochemical potential changes in different narrow ranges by involving multiple redox couples (Figure 5), it is a multistep and multielectron redox buffer.^1,2^ This thermodynamic approach does not address specific kinetic or mechanistic issues.

### Difference in the Activity and Redox Buffering Ability of **PV_6_Mo_6_** versus **PV_6_W_6_**

**Figure 8:**
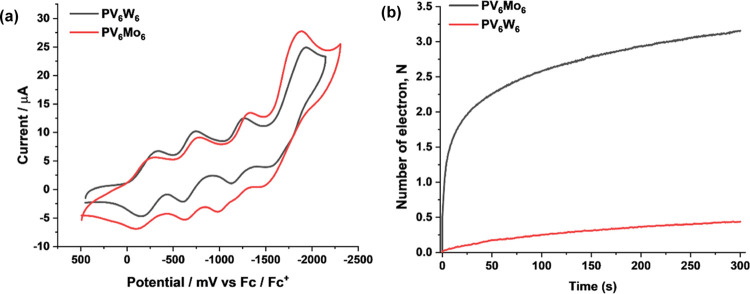
(a) Cyclic voltammograms of 0.5 mM **PV**_**6**_**Mo**_**6**_ and **PV**_**6**_**W**_**6**_ on a glassy carbon electrode in acetonitrile under argon. (b) Kinetics of **PV**_**6**_**W**_**6**_ and **PV**_**6**_**Mo**_**6**_ reduction by 25 mM RSH under Ar catalyzed by 2 μM Cu(II).

Oxidation of RSH proceeds much faster in the presence of **PV**_**6**_**Mo**_**6**_ than in the presence of **PV**_**6**_**W**_**6**_ (Table 1), yet their reduction potentials are almost the same (Figure 8a). We compared the kinetics of **PV**_**6**_**W**_**6**_ and **PV**_**6**_**Mo**_**6**_ reduction by 25 mM RSH under Ar catalyzed by 2 μM Cu(II) (Figure 8b). Half of the initial **PV**_**6**_**W**_**6**_ is reduced in 450 s, while the same process requires less than 1 s for **PV**_**6**_**Mo**_**6**_. Under turnover conditions (Figure S14), **PV**_**6**_**W**_**6**_ is reduced by one electron in 200 s independent of thiol concentration [RSH]. Over approximately the same length of time, **PV**_**6**_**Mo**_**6**_ is reduced by 2.2–3.0 electrons at a rate dependent on [RSH]. Thus, **PV**_**6**_**W**_**6**_ is unable to reach higher reduction states and thus does not exhibit a redox buffer capability like **PV**_**6**_**Mo**_**6**_. Since both POMs have almost the same CV behavior, the huge difference in catalytic activity and reduction states cannot be explained based on thermodynamics.

Figure S15 shows that Cu can catalyze the RSH reduction of **PV**_**6**_**W**_**6**_ under Ar, but only by a maximum of one electron. −d[*n*]_0_/d*t* was calculated from Figure 8b and found to be 0.47 and 8.6 × 10^–4^ s^–1^ for **PV**_**6**_**Mo**_**6**_ and **PV**_**6**_**W**_**6**_, respectively. This 3-orders-of-magnitude rate difference clearly cannot be explained by an outer-sphere electron transfer mechanism operative in all cases, given that these two reduced POMs have the same negative charges and nearly identical sizes.^79,86−88^ Indeed, previous studies show that heteropolytungstates are more likely to undergo outer-sphere electron transfer mechanisms.^79,87−91^ Neumann^92,93^ provided evidence for a generic inner-sphere electron transfer mechanism between O_2_ and PV_2_Mo_10_^5–^ and a Mars–van Krevelen-type electron transfer-oxygen transfer reaction mechanism for aerobic oxidations catalyzed by PV_2_Mo_10_^5–^.^19,94,95^ That PV_2_Mo_10_^5–^ functions via inner-sphere mechanisms in these oxidations is consistent with the hydrolytically labile Mo–O bonds.^96^ Thus, we attribute the huge activity difference and reduction state behavior difference between **PV**_**6**_**Mo**_**6**_ and **PV**_**6**_**W**_**6**_ to the electron transfer mechanism difference, inner-sphere versus outer-sphere electron transfer, respectively.

One possibility is that **PV**_**6**_**Mo**_**6**_ and **PV**_**6**_**W**_**6**_ have different types of interaction with Cu(II) ions in acetonitrile. Figure S23 shows that after half equivalent of Cu(II), the ^31^P NMR spectrum of **PV**_**6**_**Mo**_**6**_ totally disappears. The addition of 0.25 equiv of Cu(II) generates a broad peak that shifts 2.09 ppm. Bajpe et al.^97^ showed that adding Cu(NO_3_)_2_ shifts the ^31^P, ^183^W, and ^17^O NMR peaks of heteropolyacids (HPAs), such as H_3_PMo_12_O_40_ and H_3_PW_12_O_40_, in an acidic buffer. In their work, they observed clear ^31^P NMR peaks after the addition of Cu(II) ions and did not observe peak broadening. They proposed that the NMR peak shift is caused by the interaction between Cu(II) ions and the terminal oxygens of the Keggin polyanion.^97^ In the medium used for our catalytic studies here, acetonitrile, the strong perturbation of ^31^P NMR peaks suggests a very strong interaction between paramagnetic Cu(II) and vanadopolymolybdates. We established that **PVW**_**11**_ and **PV**_**6**_**W**_**6**_, unlike the corresponding vanadopolymolybdate analogues, show no peak shift and broadening after adding Cu(II) in acetonitrile (Figure S24). These results and those of Bajpe et al.^97^ are consistent with different Cu(II) speciation and POM association chemistry in acetonitrile versus water.^98^ We postulate that the dramatic effect of Cu(II) on the ^31^P NMR spectra of **PVMo** in acetonitrile likely involves covalent bond formation between POM oxygens and Cu(II), which in turn involves some displacement of acetonitrile ligands on Cu(II) by POM oxygen. This is a contact ion pair or, conveniently, an inner-sphere interaction. In contrast, there is likely little or no formation of Cu(II)–**PVW** oxygen covalent (dative) bonds, thus far less influence of the *S* = 1/2 copper centers on the central phosphorus atom and its NMR properties.

## Conclusions

(1) Polyvanadomolybdates and Cu(II) form a highly synergistic catalyst for the aerobic deodorization of a representative thiol, 2-mercaptoethanol (RSH), by oxidation to the corresponding nonodorous disulfide. Either polyvanadomolybdate or Cu alone is almost inactive. Other d-electron transition metals, with the exception of Pd, in combination with polyvanadomolybdates, are not effective aerobic oxidation catalysts.
(2) We have identified the key steps in the optimal system, TBA_4_H_5_PMo_6_V_6_O_40_ (**PV**_**6**_**Mo**_**6**_) + Cu(II). Significantly, **PV**_**6**_**Mo**_**6**_ maintains the electrochemical potential of the solution in certain narrow ranges by controlling the speciation and concentration ratio of Cu(II)/Cu(I) during catalytic turnover and, as such, constitutes a redox buffer. Under turnover conditions, several redox couples of the POM are clearly involved. This is quite distinct from the numerous other studies of organic substrate oxidations catalyzed by POMs. This phenomenon has not been previously noted in the voluminous hydrolytic and catalytic oxidation chemistry of polyvanadophosphates.
(3) Also unique to our knowledge, Cu functions as a dual catalyst in the **PV**_**6**_**Mo**_**6**_/Cu(II)/air oxidation system; Cu catalyzes the reduction of the POM by the substrate, RSH, and it also catalyzes the oxidation of the reduced POM by O_2_/air.
(4) **PV**_**6**_**W**_**6**_ does not have the redox buffer effect like **PV**_**6**_**Mo**_**6**_. As a consequence, the latter, therefore, shows no catalytic activity. Since both POM systems have almost the same electrochemical behavior, the huge electron transfer rate difference between them and Cu complexes is attributed to the inner-sphere versus outer-sphere electron transfer mechanisms.

## Supporting Information Available

Additional experimental details, materials, and methods, including electrochemical analysis, titration methods, kinetics analysis, and redox speciation calculations (PDF)
