# Supplementary material for: Catalytic System for Aerobic Oxidation That Simultaneously Functions as Its Own Redox Buffer
Source: Inorg Chem. 2023 Jan 25;62(5):2404–14. doi: 10.1021/acs.inorgchem.2c04209 (PMC9906773; doi:10.1021/acs.inorgchem.2c04209)
Supplement: Supplementary file 1 — ic2c04209_si_001.pdf [file ic2c04209_si_001.pdf]

Supporting Information

A catalytic system for aerobic oxidation that simultaneously functions as its own redox buffer

Xinlin Lu, Ting Cheng, Yurii V. Geletii* and Craig L. Hill*
Department of Chemistry, Emory University, Atlanta, GA 30322

Corresponding Authors
iguelet@emory.edu; chill@emory.edu

Materials and Methods

All chemicals were purchased from commercial sources and used without further purification. $TBA_6PV_3W_9O_{40}$, $TBA_4H_5PW_6V_6O_{40}$, $TBA_3H_3V_{10}O_{28}$ were synthesized according to the literature methods.[1, 2] The FT-IR spectra were collected on a Nicolet TM 600 FT-IR spectrometer by the attenuated total reflectance (ATR) sampling technique. UV−vis spectra were measured with an Agilent 8453 spectrophotometer equipped with a diode array detector using a 1.0 cm optical path length quartz cuvette. ^1H nuclear magnetic resonance (NMR) spectra were acquired on a Varian INOVA 400 spectrometer. The kinetics were followed by an SF-61 stopped-flow instrument (Hi-Tech Scientific, U.K.). Elemental analyses (N and P) were conducted by Galbraith Laboratories (Knoxville, TN). The thermogravimetric (TGA) data were collected on a Mettler Toledo TGA instrument.

Electrochemistry

Cyclic voltammograms (CVs) and bulk electrolysis data were obtained using a BAS CV-50W electrochemical analyzer and conducted at room temperature (25 ± 2 °C). CVs were recorded in a standard three-electrode electrochemical cell with a glassy carbon disk working electrode, a platinum wire counter electrode and a Ag/Ag^+ (0.01 M $AgNO_3$ in CH_3CN) reference electrode using 0.1 M tetrabutylammonium hexafluorophosphate (n-Bu_4NPF_6) as the supporting electrolyte. The scan rate used in voltametric experiments was 100 mV s^{-1}. The measured potential was converted to the Fc/Fc^+ scale using data measured from CV for 1.0 mM ferrocene (Fc). A reticulated vitreous carbon working electrode was used as a working electrode in bulk electrolysis and the working and counter electrode were separated by porous glass sinters. Each electrolysis was conducted at the desired constant potential until the current dropped to <10% of the initial value, then aliquots were withdrawn and the UV-Vis spectra were recorded under Ar. The electrolysis was then resumed at the more negative potential as listed in Table S1.

Number of electrons transferred during bulk electrolysis was calculated by Faraday's law of electrolysis $Q = nFN$, where Q is the number of coulombs, $F = 96485\ C\ mol^{-1}$ is Faraday's constant, N is the moles of substrate electrolyzed and n is the stoichiometric number of electrons consumed.

Rotating disk electrode (RDE) voltammetry was conducted on a Wavedriver 10 potentiostat/galvanostat (Pine Research Instrumentation) using a standard three electrode setup with a 3 mm diameter glassy carbon disk working electrode, a Ag/Ag^+ (0.01 M $AgNO_3$ in CH_3CN)

reference electrode, and a platinum wire counter electrode. The rotation speed was controlled by a Model AFMSRCE ring-disk electrode system (Pine Research Instrumentation).

RDE data were analyzed by Levich plots (limiting current i_L versus $\omega^{1/2}$, where $\omega^{1/2}$ is the rotation speed) using the Levich equation $i_L = 0.62nFAD^{2/3}\omega^{1/2}v^{-1/6}C_0$, where n is the number of transferred electrons in the redox reaction, $F = 96485\ C\ mol^{-1}$ is Faraday's constant, A is the area of electrode surface, v is the kinematic viscosity of the 0.1 M n-Bu_4NPF_6 acetonitrile solution and C_0 is the bulk concentration of POM.

Synthesis of TBA salts of $PMo_{12-n}V_nO_{40}^{(3+n)-}$ (n = 1-6)

$H_3PMo_{12}O_{40}$, $H_4PVMo_{11}O_{40}$, $H_5PV_2Mo_{10}O_{40}$, $H_6PV_3Mo_9O_{40}$, $(NH_4)_7PV_4Mo_8O_{40}$ and $(NH_4)_5H_4PMo_6V_6O_{40}$ were synthesized according to the literature methods.[3-7] The tetra-*n*-butylammonium (TBA) salts of POMs were precipitated from aqueous solutions with TBA bromide. $TBA_3PMo_{12}O_{40}$ (**PMo_{12}**) was prepared in 0.1 M $HClO_4$, while $TBA_4PVMo_{11}O_{40}$ (**$PVMo_{11}$**) and $TBA_4HPV_2Mo_{10}O_{40}$ (**PV_2Mo_{10}**) were prepared in 0.1 M sulfate buffer at pH 2.0. $TBA_4H_2PV_3Mo_9O_{40}$ (**PV_3Mo_9**), $TBA_4H_3PV_4Mo_8O_{40}$ (**PV_4Mo_8**) and $TBA_4H_5PMo_6V_6O_{40}$ (**PV_6Mo_6**) were prepared directly in DI water. TBA salts of the POMs were recrystallized twice in acetonitrile. The purity was confirmed by ATR FT-IR and UV-Vis spectra (Figure S16 and S17).

The FT-IR spectrum: 1480-1370 cm^{-1} can be assigned to C-H bending from the TBA counterion. **PMo_{12}**: 1059, 949, 872 and 782 cm^{-1}. **$PVMo_{11}$**: 1078, 1054, 982, 938, 865 and 787 cm^{-1}. **PV_2Mo_{10}**: 1077, 1063, 1048, 984, 935, 867, 780 cm^{-1}. **PV_3Mo_9**: 1077, 1062, 1048, 1004, 966, 935, 870 and 777 cm^{-1}. **PV_4Mo_8**: 1064, 1001, 961, 936, 876 and 765 cm^{-1}. **PV_6Mo_6**: 1062, 1001, 955, 933, 876 and 765 cm^{-1}. The peaks in the range of 1070-1030 cm^{-1} are assigned to P-O vibrations. The splitting from a signal peak is due to the positional isomers of the multi-vanadium Keggin complexes. Peaks in the range of 1000-910 cm^{-1} are assigned to M-O vibrations (where M = V or Mo), and peaks around 870 and 780 cm^{-1} are assigned to inter-octahedral M-O-M and intra-octahedral M-O-M vibrations respectively.[3, 5, 6]

The UV-Vis spectrum: 350-500 nm is attributed to the ligand-to-metal charge-transfer (LMCT) band of V(V) center, which indicates the substitution of Mo(VI) with V(V). The intensity of this band increases with the increasing number of vanadium atoms in POM as shown in Figure S17.[2]

The number of TBA counterions of the most effective catalyst in this paper, $TBA_4H_5PMo_6V_6O_{40}$, **PV_6Mo_6**, was determined by elemental analyses: N/P = 4.05. The number of TBA counterions for all the $PMo_{12-n}V_nO_{40}^{(3+n)-}$ isomers was confirmed by TGA (Figure S18), 36.37%, 36.7%, 36.91% 38.5% and 38.31% weight loss for $TBA_4PVMo_{11}O_{40}$, $TBA_4HPV_2Mo_{10}O_{40}$, $TBA_4H_2PV_3Mo_9O_{40}$, $TBA_4H_3PV_4Mo_8O_{40}$ and $TBA_4H_5PMo_6V_6O_{40}$, respectively. None of these POMs show marked loss of hydration water molecules. The slow weight loss after 400 °C is due to the thermal instability of the **PVMo** structure at and above this temperature.

Quantification of RSH

The RSH concentration was determined using Ellman's reagent (5,5-dithiobis(2-nitrobenzoic acid) (DTNB)).[8] DTNB quickly reacts with thiols through the following reaction to form 2-nitro-5-thiobenzoic acid (TNB), which absorbs in the UV-vis region at λ_{max} = 412 nm (ε = 13.1 ± 0.9x10^3 M^{-1} cm^{-1}).

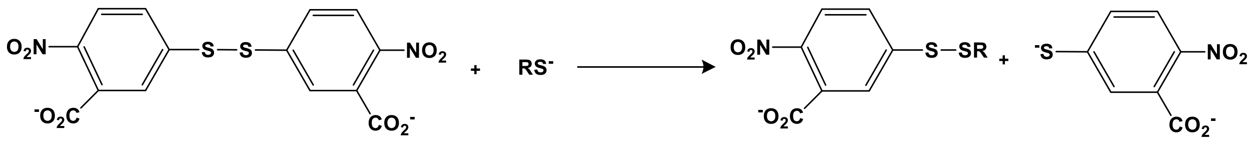

In a typical reaction, 0.1 mL of DTNB stock solution (5 mg/mL in methanol) was added to 5 mL of a 50 mM phosphate buffer solution (pH 7.4). This solution was used as a blank for UV-vis measurements. Then, 10 μL aliquot of the reaction solution was added and the absorbance at 412 nm was measured.

Oxidation of RSH

In a typical reaction, **PV_6Mo_6** (0.1 mM), $Cu(ClO_4)_2$ (0.5 mM) and 2-mercaptoethanol (30 mM) were stirred in acetonitrile under air in air-conditioned room at 25±2 °C. The aliquots of the solution were withdrawn every several minutes and monitored as discussed above.

Reduction State of PV_6Mo_6 under Steady State Conditions

In a typical experiment, **PV_6Mo_6** (0.1 mM) and $Cu(ClO_4)_2$ (0.5 mM) were stirred in acetonitrile purged with air in a 1.0 cm optical path length quartz cuvette at 25±2 °C. The changes in the UV-Vis spectra of the solution in the course of the reaction were monitored after adding the 2-mercaptoethanol (30 mM). The absorption was then converted to the apparent extinction coefficient using the Beer–Lambert law. The average number of electrons transferred to the POM was calculated from the calibration curve in Figure S6.

Stopped-Flow Measurements

The rates of $PV_nMo_{12-n}O_{40}^{(3+n)-}$ reduction by 2-mercaptoethanol at different concentrations of $Cu(ClO_4)_2$ [Cu(II)] were measured under argon by recording changes in visible spectra using the stopped-flow technique. In a typical experiment, one feeding syringe was filled with the de-aerated stock acetonitrile solution of POM and Cu(II). The second syringe was filled with the de-aerated acetonitrile solution of 2-mercaptoethanol. In all stopped-flow kinetic measurements, the concentrations of POM, Cu(II) and 2-mercaptoethanol in the reaction mixture were two times lower than those in the feeding syringes.

Reoxidation of Reduced POMs by O_2.

PV_6Mo_6 was reduced by 3 equivalents (6-electron reduction) of ascorbic acid and kept under argon. Reoxidation was followed by UV-vis absorbance on 550 nm. In a typical experiment, after adding the Cu(II) stock solution to the 6-electron reduced POM solution in a 1.0 cm optical path length quartz cuvette, the O_2 was purged through the solution, and the absorbance was monitored as a function of time.

Reaction Stoichiometry Determination

The stoichiometry was determined by monitoring the oxygen consumption using a pressure monometer (Figure S1). In a typical experiment, **PV_6Mo_6** (0.1 mM), $Cu(ClO_4)_2$ (0.8 mM) and 2-mercaptoethanol (15 mM, 30 mM) was stirred in acetonitrile (30 mL) in a double-neck, jacketed glass flask (90 mL). The pressure monometer was connected to the flask and the system was airtight. The pressure drop due to the oxygen consumption was read by pressure monometer in mm Hg units. The volume of head space was 57 mL, and the solution volume was 30 mL.

The pressure dropped by 68 mm Hg when the RSH concentration was 30 mM, 0.895 mmol. The molar ratio between consumed RSH and consumed oxygen calculated from eq 1 and eq 2 was 4.06 ± 0.08. Similarly, the pressure dropped by 35 mm Hg in the reaction of RSH (15 mM, 0.447 mmol) when the molar ratio of RSH to oxygen was 3.96±0.08, which can be calculated from eq 3 and eq 4. In addition, the pressure drop was 35 mm Hg at 15 mM RSH, roughly a half the 68 mm Hg drop at 30 mM RSH, which confirms the stoichiometry of the reaction.

$$\Delta 68mmHg = \Delta\, 9.57kpa \qquad (1)$$

$$\Delta pv = \Delta nRT \; ; \Delta n = \frac{9.57kpa \times 0.057L}{8.3145\times(24+273.15)} = 2.24\times10^{-4};\; \frac{8.95\times10-4}{2.24\times10-4} = 4.06 \qquad (2)$$

$$\Delta 35mmHg = \Delta\, 4.93kpa \qquad (3)$$

$$\Delta n = \frac{4.93kpa \times 0.057L}{8.3145\times(24+273.15)} = 1.13\times10^{-4};\; \frac{4.47\times10-4}{1.13\times10-4} = 3.96 \qquad (4)$$

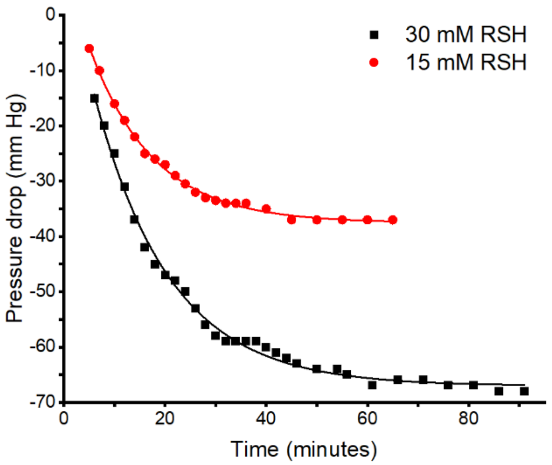

Figure S1. Oxygen consumption curve. Solid points: real experimental data. Solid line: exponential fitting curve.

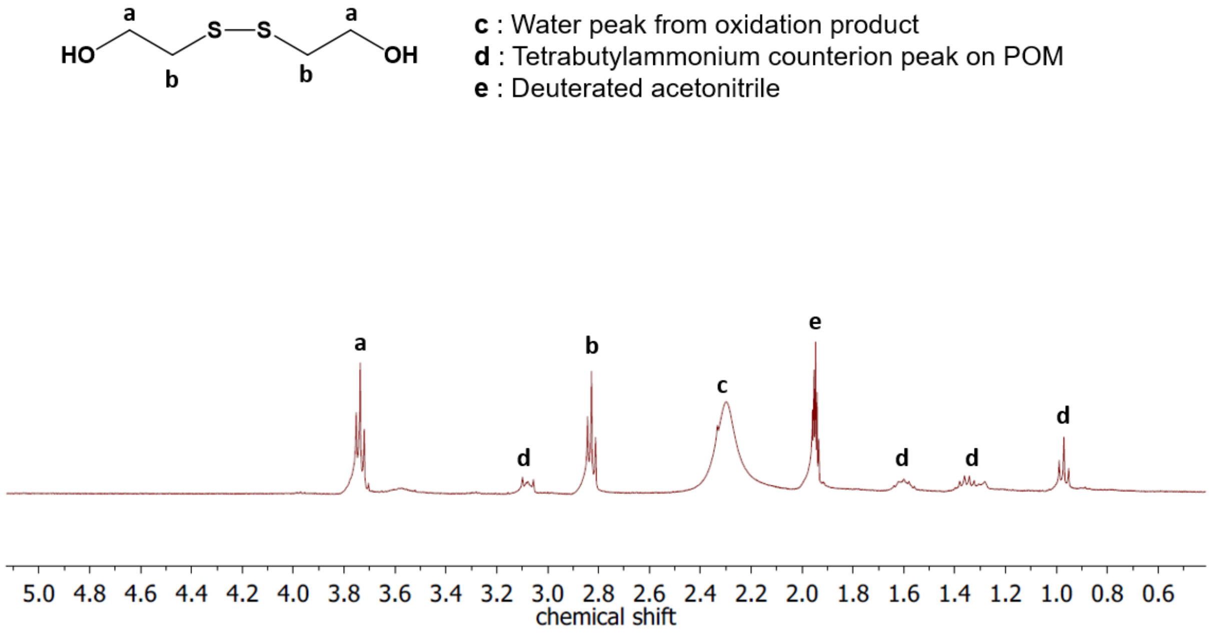

Figure S2. ^{1}H NMR of the solution at the end of the reaction: confirmation that the product of 2-mercaptoethanol oxidation is bis(2-hydroxyethyl) disulfide. Conditions: **PV_6Mo_6** (0.1 mM), $Cu(ClO_4)_2$ (0.8 mM) and 2-mercaptoethanol (30 mM) stirred in deuterated acetonitrile (5 mL) under air.

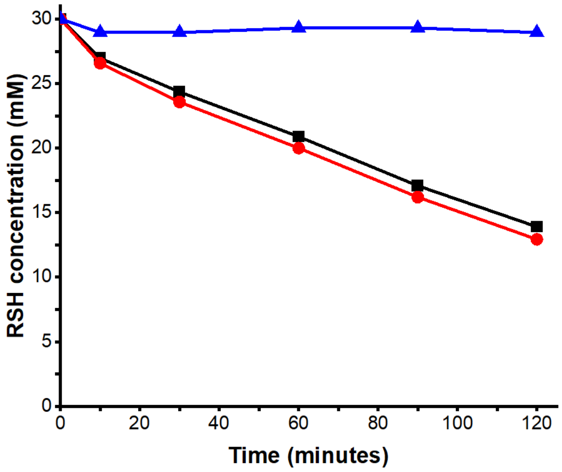

Figure S3. Kinetics of 2-mercaptonethanol oxidation by air. Black curve: **PV_6Mo_6** (0.1 mM) with $Cu(ClO_4)_2$ (0.5 mM); red curve: **PV_6Mo_6** (0.1 mM), $NaVO_3$ (0.1 mM) with $Cu(ClO_4)_2$ (0.5 mM). Blue curve: $NaVO_3$ (0.5 mM) with $Cu(ClO_4)_2$ (0.5 mM). Conditions: 2-mercaptoethanol (30 mM) in acetonitrile (5 mL). $NaVO_3$ was dissolved in hot DI water to make a stock solution. Stock solution (50 µL) was added to the acetonitrile solution resulting in 0.1 mM $NaVO_3$. Since $NaVO_3$ does not dissolve in acetonitrile, an aqueous stock solution of $NaVO_3$ with the required concentration was added to the acetonitrile solution.

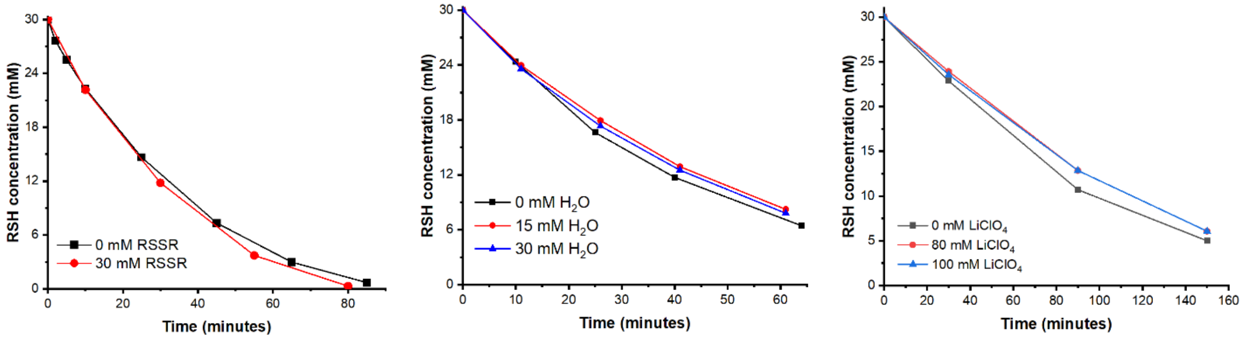

(a) (b) (c)

Figure S4. Effects of the reaction products and ionic strength on kinetics of 2-mercaptonethanol oxidation by air. (a) **RSSR effect: PV_6Mo_6** (0.1 mM), $Cu(ClO_4)_2$(0.5 mM) and 2-mercaptoethanol (30 mM). Black curve: bis(2-hydroxyethyl) disulfide (RSSR) (0 mM initial concentration); red curve: RSSR (30 mM initial concentration). (b) **H_2O effect: PV_6Mo_6** (0.1 mM), $Cu(ClO_4)_2$ (0.5 mM) and 2-mercaptoethanol (30 mM). Black curve: H_2O (0 mM); red curve: H_2O (15 mM); blue curve: H_2O (30 mM). (c) **Ionic strength effect by varying $LiClO_4$ concentration: PV_6Mo_6** (0.2 mM), $Cu(ClO_4)_2$ (0.5 mM) and 2-mercaptoethanol (30 mM). Black curve: $LiClO_4$ (0 mM); red curve: $LiClO_4$ (80 mM); Blue: $LiClO_4$ (100 mM).

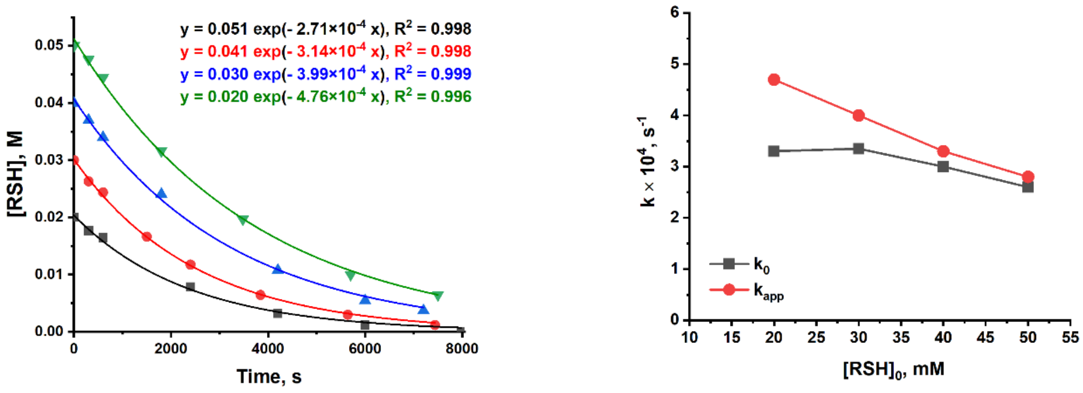

(a) (b)

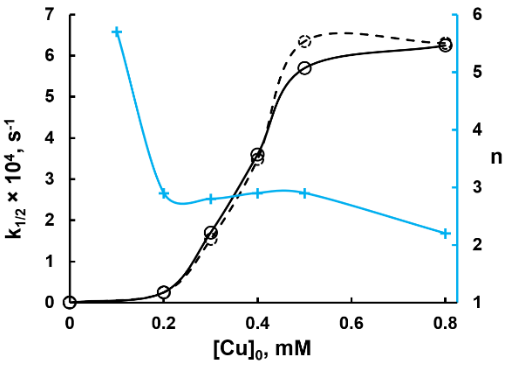

(c)

Figure S5. (a) Kinetics of RSH consumption versus time. Solid lines: the fitting to the equation $[RSH] = [RSH]_0exp(-k_{app}t)$. Conditions: **$PV_6Mo_6$** (0.1 mM) and $Cu(ClO_4)_2$ (0.5 mM). (b) red: k_{app}; black: k_0 from fitting to the equation $[RSH] = [RSH]_0(exp(-k_0t) – k_1t)$; (c) 0.1 mM **$PV_6Mo_6$** and 30 mM RSH, black solid and dashed: $k_{1/2}$ and $1.45k_0$, respectively; blue: the reduction state of **PV_6Mo_6** expressed in n.

After some optimization, we chose the following range of initial concentrations (in mM) for our detailed studies: 20-50 for RSH, 0.025-0.8 for POM, **PV_6Mo_6**, and 0.1-1.0 for Cu(II). The approach to measure the initial rates did not work effectively, because of low accuracy in measurement of initial rates. Under the conditions in Figure 1, the consumption of RSH fits well to the exponential curve, $[RSH] = [RSH]_oexp(-k_{app}t)$. However, the values of k_{app} depends on $[RSH]_0$ (Figure 2b), which intrinsically contradicts the exponential law. We found that all kinetic curves can be fitted to the mechanism, which includes a combination of the zero and the first order pathways: $[RSH] = [RSH]_o\{exp(-k_0t) - k_1t\}$. If the contribution of the zero order is comparable with that of the first order, the question arises how to quantify the catalytic activity of such systems. Therefore, we measured the time required to reach 50% conversion of RSH, $t_{1/2} = 1/k_{1/2}$. If the first order pathway is dominant, then $k_{1/2} = ln(2)k_0 \approx 1.45\ k_0$. The difference between k_{app} and k_0 is small, but results in the weak dependence k_{app} on $[RSH]_0$. The difference between $1.45\ k_0$ and $k_{1/2}$ is commonly small as shown on Figure S5c. All these make reasonable to use $k_{1/2}$ as a measure of catalytic activity.

The highest TON and TOF were achieved at 0.1 mM **PV_6Mo_6** and 0.5 mM Cu(II) (Table 1). Therefore, we have chosen these concentrations as the starting points for detailed studies. An increase of $[RSH]_0$ from 20 to 50 mM results in a weak decrease in k_{app}, while k_0 remains almost constant (Figure S5b). The dependence of catalytic activity on Cu(II) expressed as $k_{1/2}$ has an S-shape (Figure S5c). The two $k_{1/2}$ values agree with each other indicating that the first order pathway dominates during this Cu concentration range.

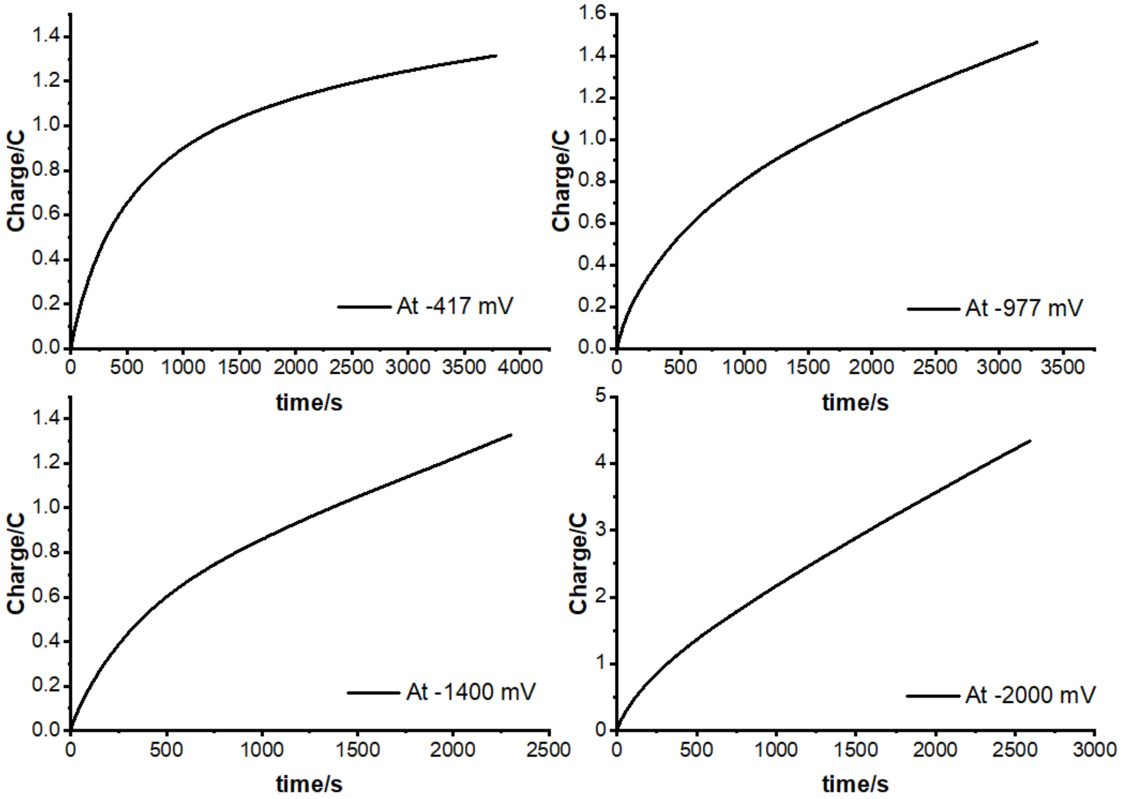

Figure S6. Charge vs time curves from the bulk electrolysis of **PV_6Mo_6**. Conditions: **PV_6Mo_6** (0.5 mM), *n*-Bu_4NPF_6 (100 mM) in acetonitrile (30 mL), at 25±2 °C under argon. See Table S1 below.

Table S1. Bulk electrolysis of PV_6Mo_6 at constant potentials in acetonitrile.[a]

Potential/mV vs Fc/Fc^+	Number of coulombs	Number of electrons accumulated by POM[b]	Ending current ratio/%
-417	1.32	1	1.8
-977	1.46	2	4.4
-1400	1.4	3	5
-2000	2.8	5	12

[a] Conditions: **PV_6Mo_6** (0.5 mM), *n*-Bu_4NPF_6 (100 mM), acetonitrile (30 ml), at room temperature under argon. [b] Total number of electrons transferred, calculated according to Faraday's law of electrolysis.

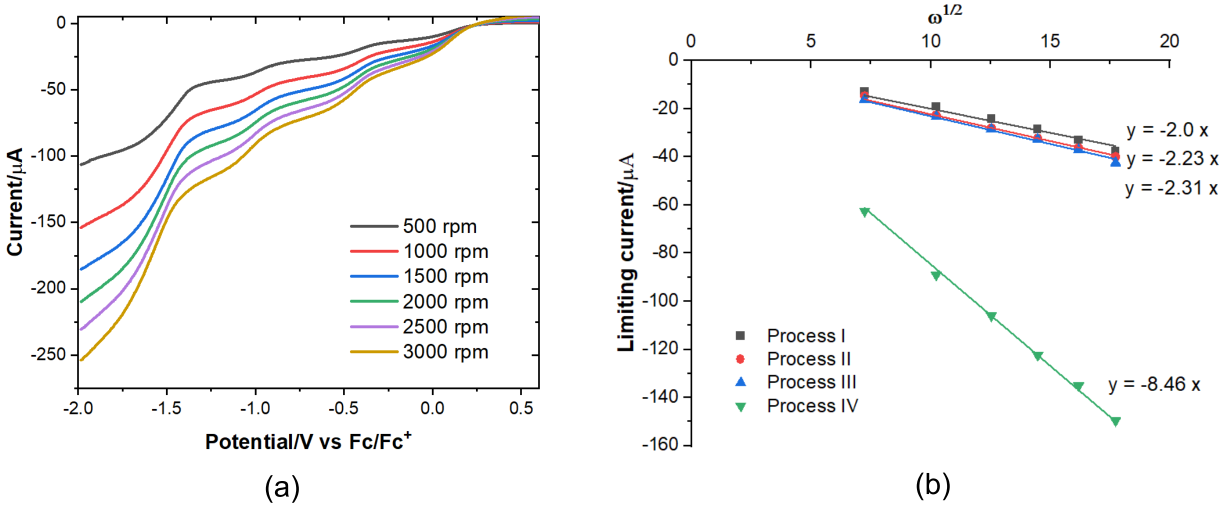

Figure S7. (a) Rotating disk electrode voltammetric raw data at different electrode rotation speeds from 500 rpm to 3000 rpm. (b) Levich plot for all four processes. Conditions: **PV_6Mo_6** (0.5 mM), *n*-Bu_4NPF_6 (100 mM), acetonitrile (20 ml), at room temperature under argon, scan rate 5 mV s^{-1}

Table S2. $E_{1/2}$ from rotating disk electrode voltammetric measurements.[a]

	Process I	Process II	Process III	Process IV
$E_{1/2}$/mV vs Fc/Fc^+	-202	-721	-1285	-1810

[a] $E_{1/2}$ was measured by averaging all rotation speeds. Conditions: **PV_6Mo_6** (0.5 mM), *n*-Bu_4NPF_6 (100 mM), acetonitrile (20 ml), at room temperature under argon, scan rate = 5 mV s^{-1}

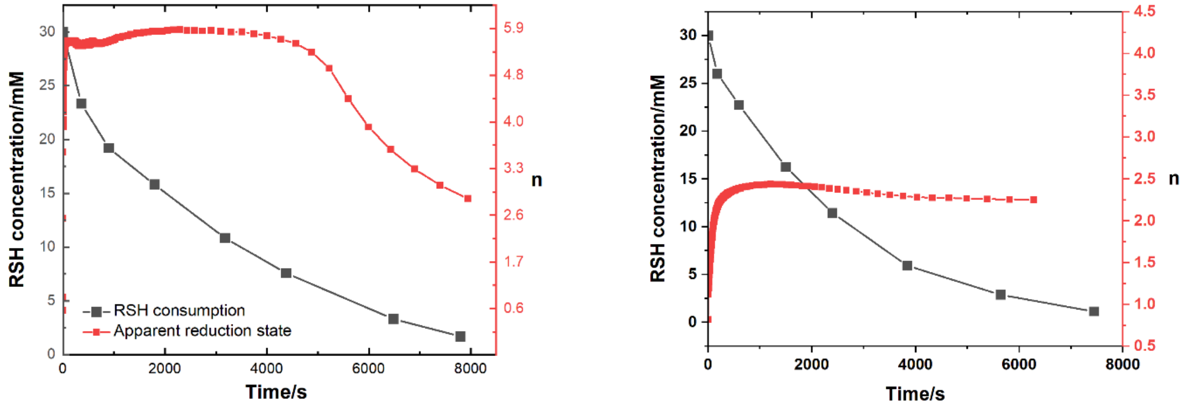

(a) (b)

Figure S8. Black: The kinetics of 2-mercaptoethanol (30 mM) oxidation in the presence of $Cu(ClO_4)_2$ (0.5 mM), (a) **PV_6Mo_6** (0.6 mM), (b) **PV_6Mo_6** (0.1 mM). Red: Apparent reduction state, n, of **PV_6Mo_6** at 550 nm.

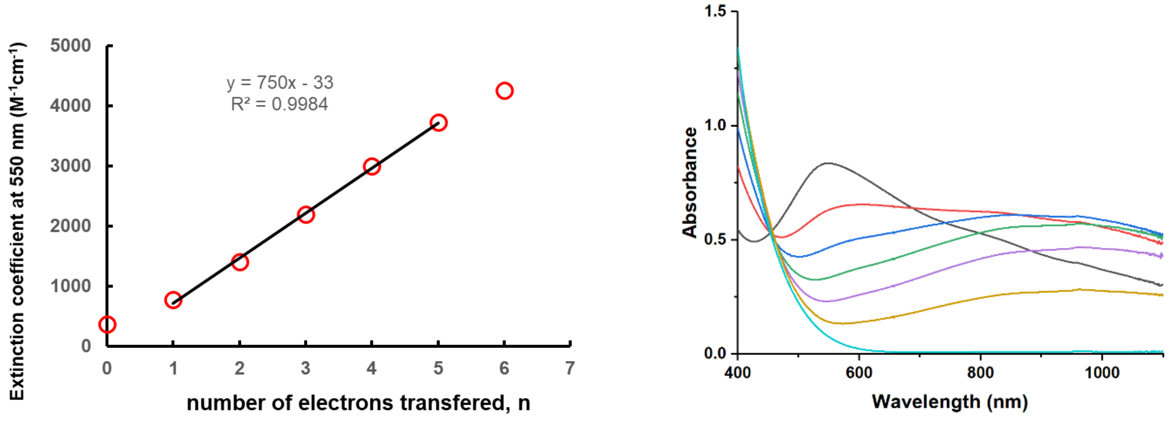

(a) (b)

Figure S9. (a) Titration of **PV_6Mo_6** by ascorbic acid in acetonitrile under Ar. (b) Extended UV-Vis spectra resulting from ascorbic acid titration.

Redox speciation and potentials of PV_6Mo_6

The POM distribution in different reduction states depends on chemical solution potential E and is described by eq S5, where E_i is the standard reduction potential of a (**PV_6Mo_6**)$_i$/(**PV_6Mo_6**)$_{i+1}$ couple measured electrochemically

$$\alpha_i = (\alpha_{i-1}10^{(Ei - E)/60})/(\textstyle\sum_{i=0}^{i=6} \alpha i) \qquad (5)$$

The apparent reduction state of POM, n, is calculated by eq 5, and the results are given in Figure S9.

$$n = \textstyle\sum_0^6 i(\alpha i) \qquad (6)$$

The speciation of highly reduced **PV_6Mo_6** (n > 3) depends on reduction potentials E_5 and E_6, which are not known. Exemplary results assuming E_5 = E_6 = -1500 mV are given in Figure 5. The reduction states of **PV_6Mo_6** measured under different reaction conditions all fall into the range of n = 2 to 3 and 3 to 6 which correspond to the potential range of E = -(1180-1340) and -(1600-1680) mV, respectably.

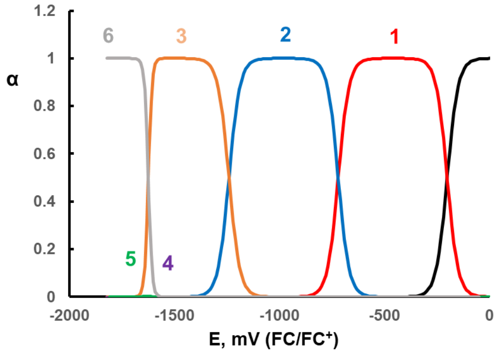

Figure S10. The distribution diagram of the reduced forms of **PV_6Mo_6** as a function of chemical solution potential.

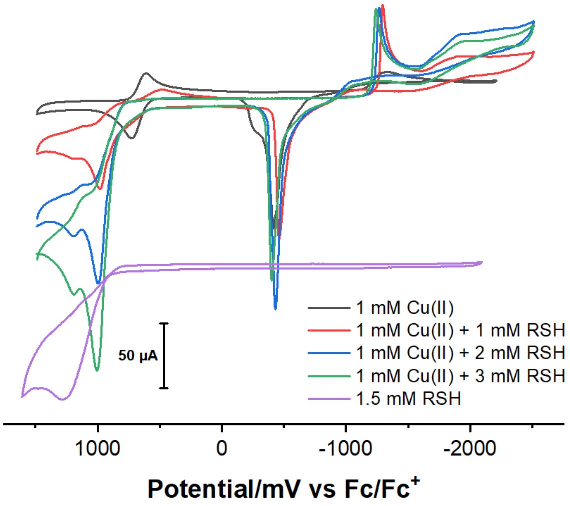

Figure S11. Cyclic voltammograms (CV) of 1.0 mM $Cu(ClO_4)_2$ with different concentrations of 2-mercaptoethanol. Conditions: 100 mM *n*-Bu_4NPF_6, scan rate 100 mV s^{-1}, T = 298 K. The peak at 675 mV has an anodic-cathodic peak potential separation of 130 mV and the ratio of currents is close to 1 in the positive potential domain. This peak is assigned to the reversible Cu(II)/Cu(I). The potential is on the high side of the range for this couple in other complexes, but in agreement with the literature value 950 mV versus SCE. The large difference between anodic and cathodic potentials is consistent with a sluggish electron transfer from Cu to the electrode. In the negative potential domain, the CVs are difficult to interpret due to deposition of Cu(0) and adsorption of RSH on electrode. With two equivalents of RSH, the Cu(II)/Cu(I) peak totally disappears, which proves the formation of a complex between Cu and RSH. The newly generated peaks at negative potential may belong to the complexes Cu(II)RSH and $Cu(II)(RSH)_2$. One of the peaks around +1000mV is the RSH peak confirmed by comparing with the CV of RSH alone.

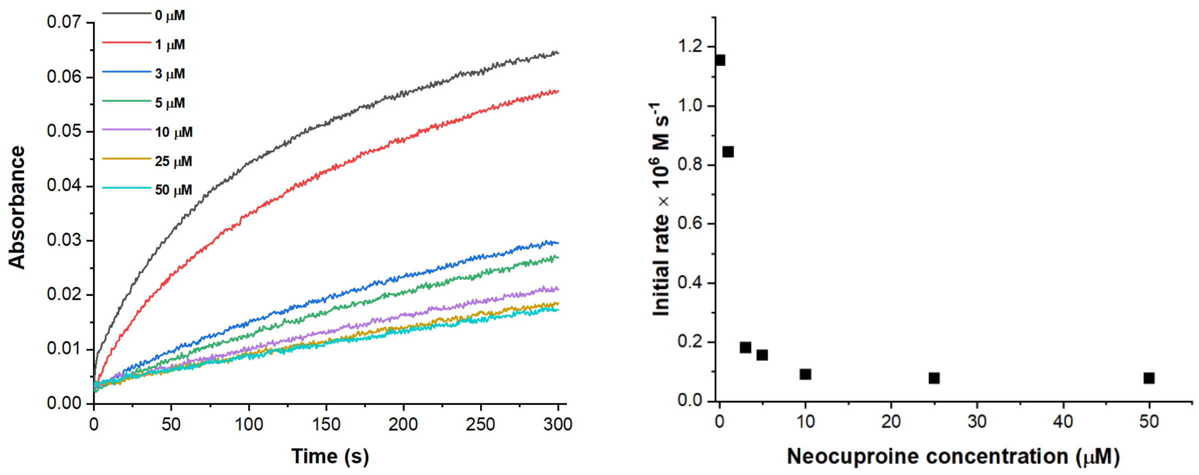

(a) (b)

Figure S12. (a) Stopped-flow kinetics data for **PV_6Mo_6** reduction by 2-mercaptoethanol at different neocuproine concentrations. (b) Initial rates versus neocuproine concentration. Conditions: **PV_6Mo_6** (0.4 mM), 2-mercaptoethanol (50 mM), acetonitrile, under argon.

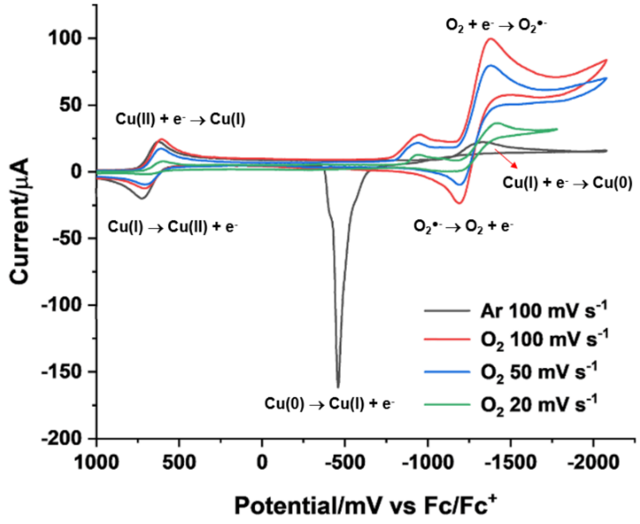

Figure S13. CV of 1 mM $Cu(ClO_4)_2$ under argon and O_2 with different scan rates. Conditions: 100 mM *n*-Bu_4NPF_6, T = 298 K.

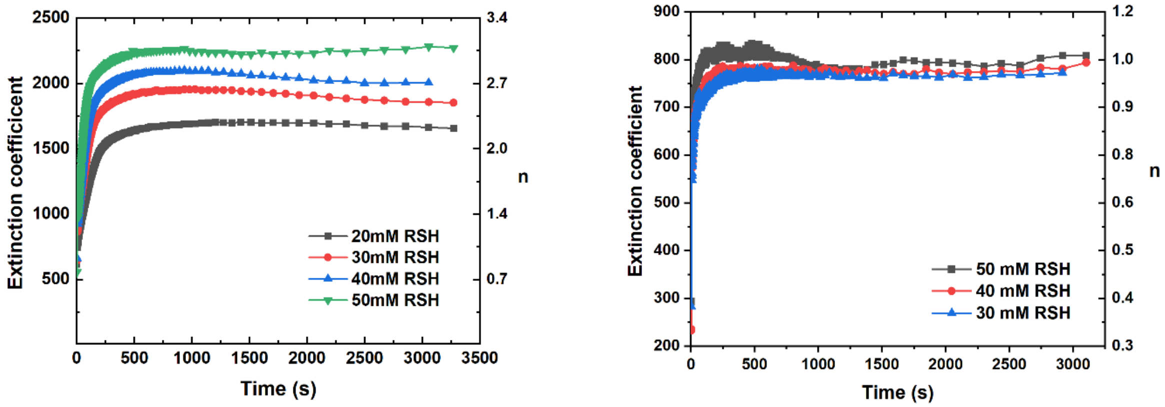

(a) (b)

Figure S14. Apparent reduction state of (a) **PV_6Mo_6** and (b) **PV_6W_6** depends on RSH concentration. Conditions: POM (0.1 mM), Cu(II) (0.5 mM) under air in acetonitrile.

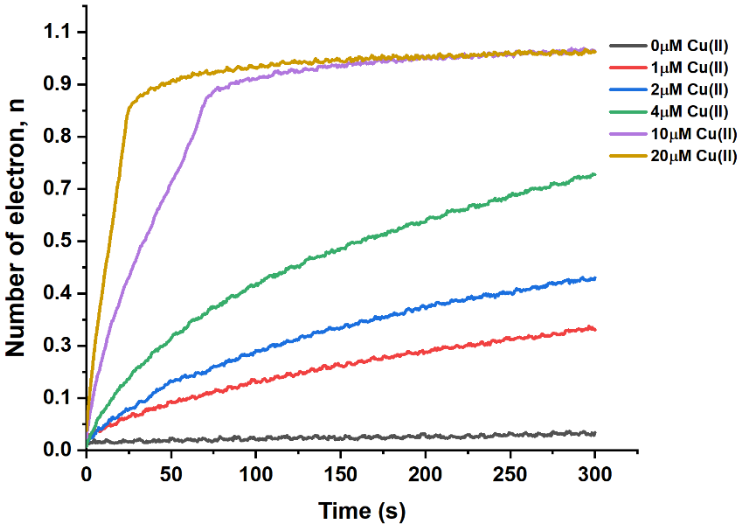

Figure S15. Cu(II) dependence of **PV_6W_6** reduction by RSH under Ar. Conditions: 25 mM RSH, 0.5 mM **PV_6W_6** in acetonitrile in room temperature.

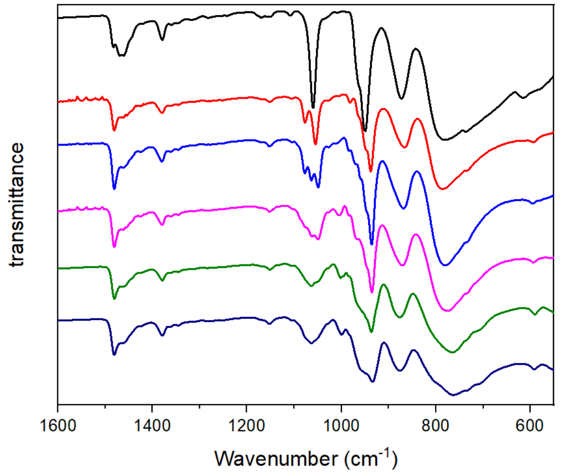

Figure S16. FT-IR (ATR) spectra. $TBA_3PMo_{12}O_{40}$ (black), $TBA_4PVMo_{11}O_{40}$ (red), $TBA_4HPV_2Mo_{10}O_{40}$ (blue), $TBA_4H_2PV_3Mo_9O_{40}$ (pink), $TBA_4H_3PV_4Mo_8O_{40}$ (green) and $TBA_4H_5PMo_6V_6O_{40}$ (TBA_4H_5**PV_6Mo_6**; dark blue).

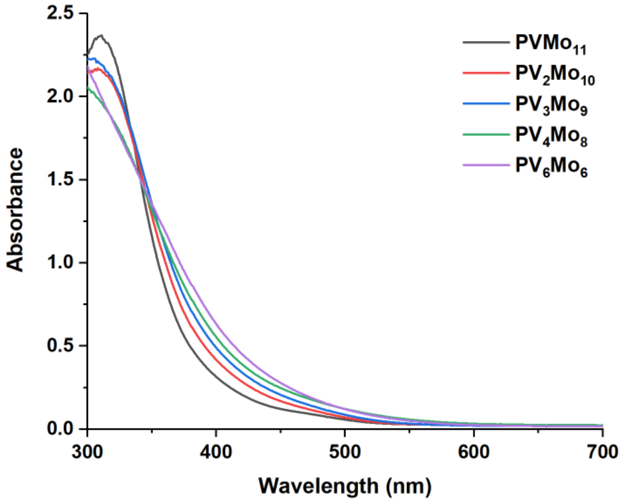

Figure S17. UV-vis spectra of TBA salts of $PV_nMo_{12-n}O_{40}^{(3+n)-}$.

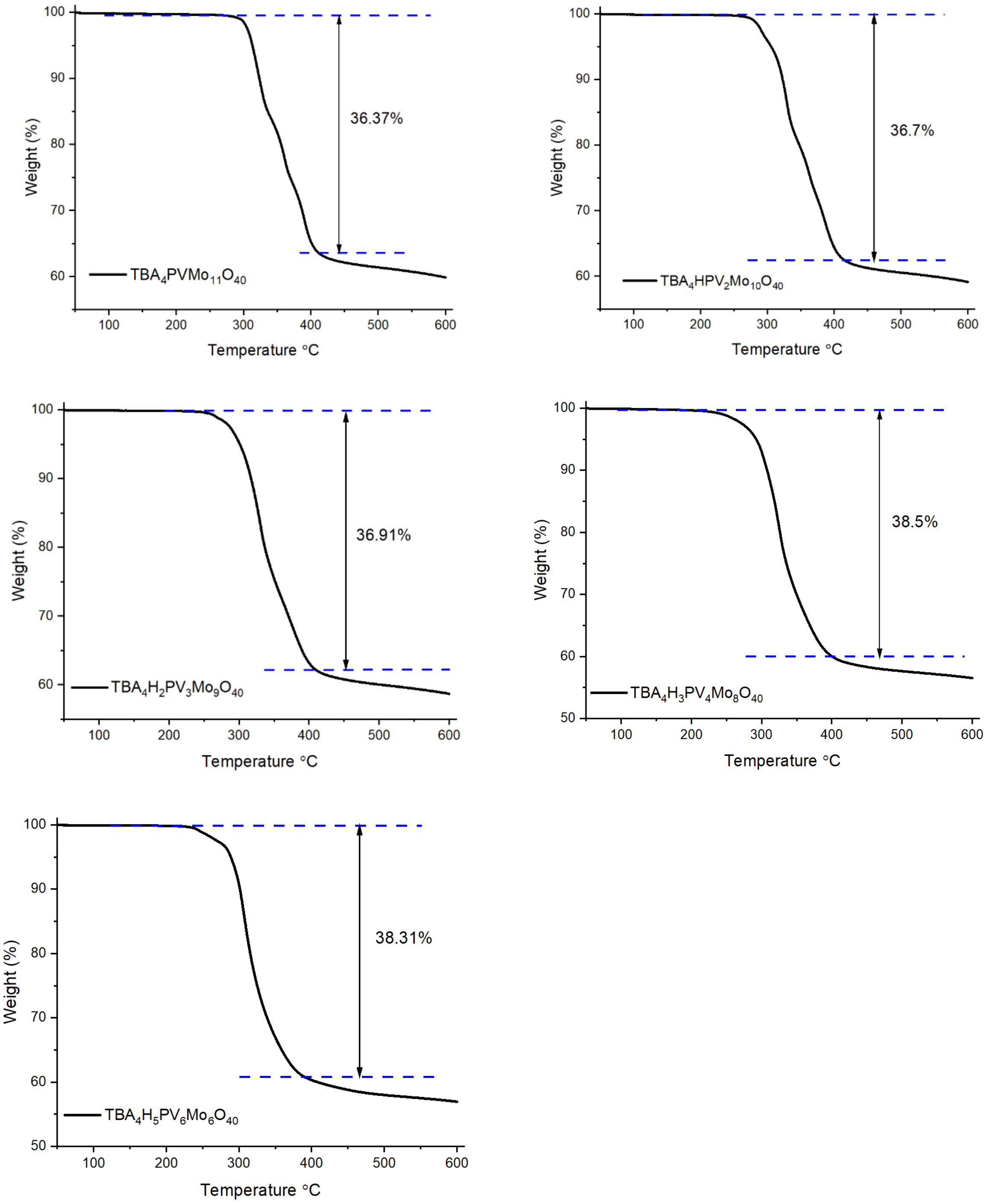

Figure S18. Thermogravimetric analysis (TGA) data of TBA salts of $PV_nMo_{12-n}O_{40}^{(3+n)-}$.

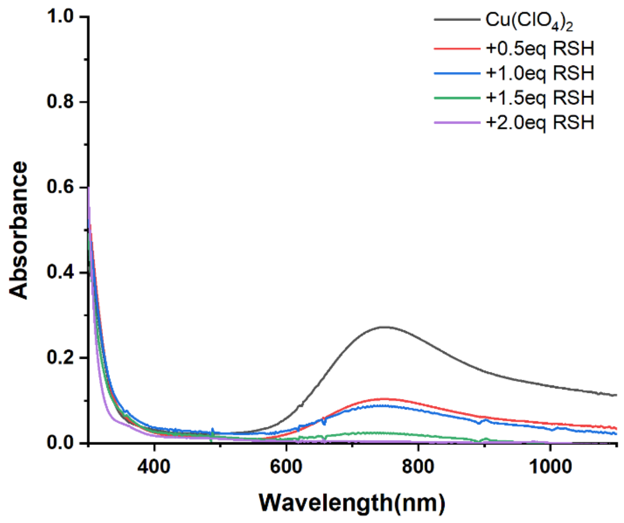

Figure S19. 2-mercaptoethanol titration $Cu(ClO_4)_2$ in acetonitrile. Experiment done in a 10mL UV-Vis cuvette with 0.1mM $Cu(ClO_4)_2$ in 25mL acetonitrile. Titration is monitored at 750nm peak of Cu^{2+} ion.

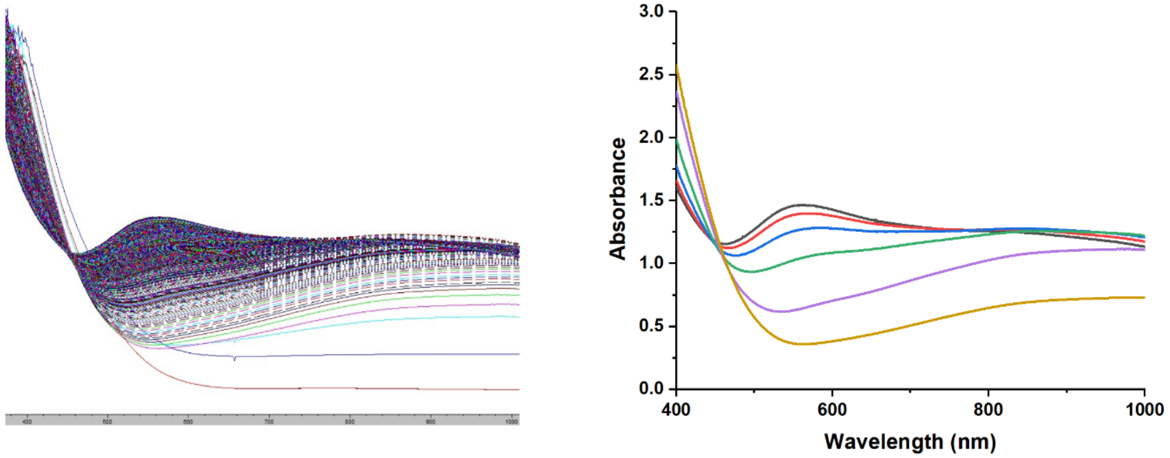

(a) (b)

Figure S20. (a) Screen shot of the UV-Vis spectra of **PV_6Mo_6** kinetics during catalytic turnover. (b) Plot of selected spectra from (a) for clarity.

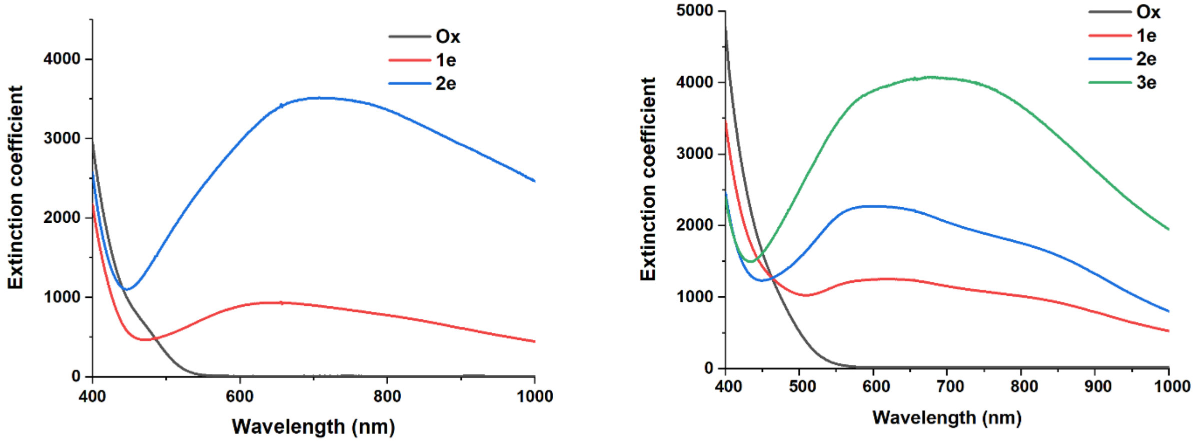

(a) (b)

Figure S21. UV-Vis spectra from bulk electrolysis titration of (a) **$PVMo_{11}$**; (b) **PV_2Mo_{10}**.

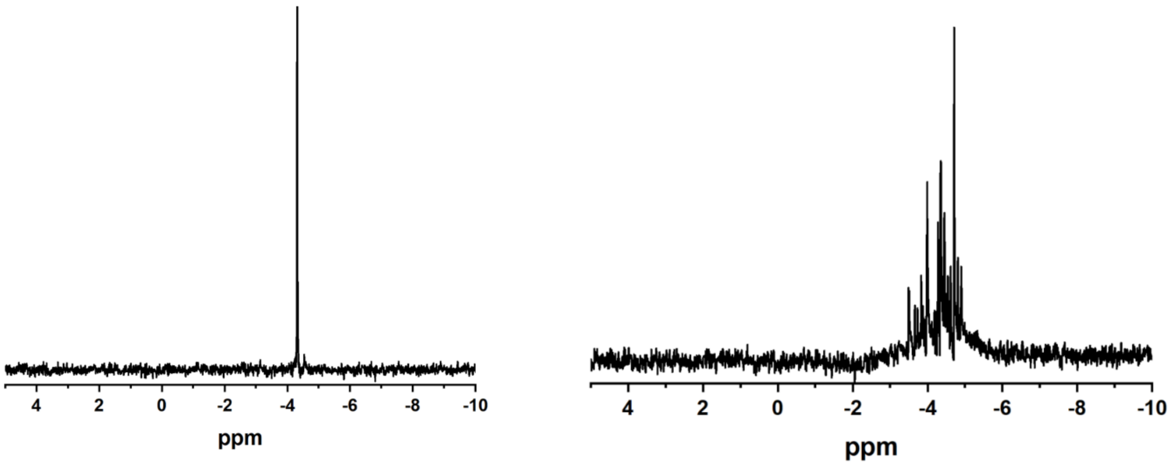

(a) (b)

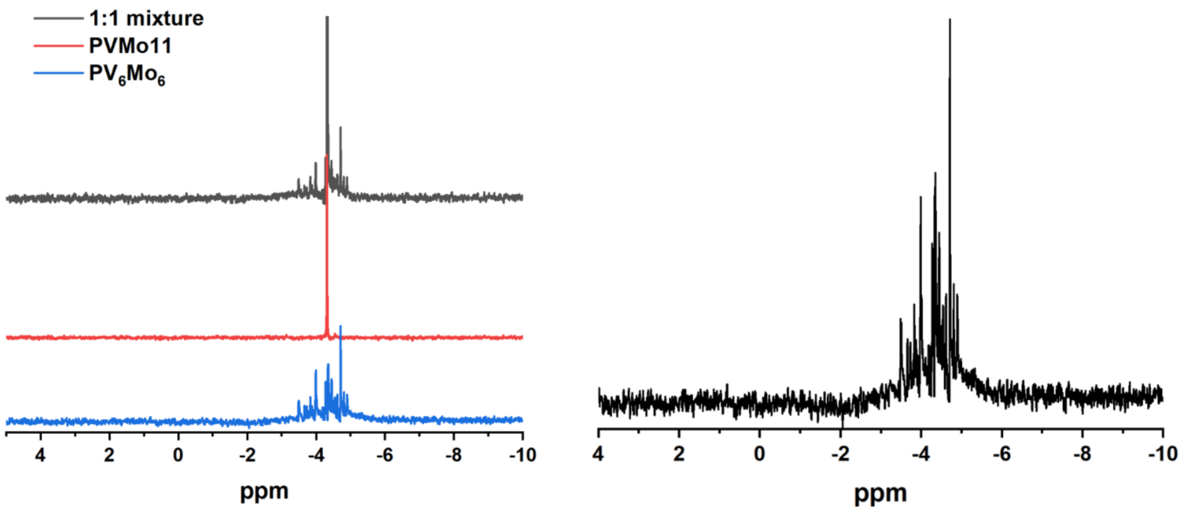

(c) (d)

Figure S22. ^{31}P NMR spectra in acetonitrile-d$_3$ with respect to 85% H_3PO_4 (0 ppm) (a) **$PVMo_{11}$**; (b) **PV_6Mo_6**; (c) 1:1 mixture of **$PVMo_{11}$** and **PV_6Mo_6**; (d) **PV_6Mo_6** after aging overnight.

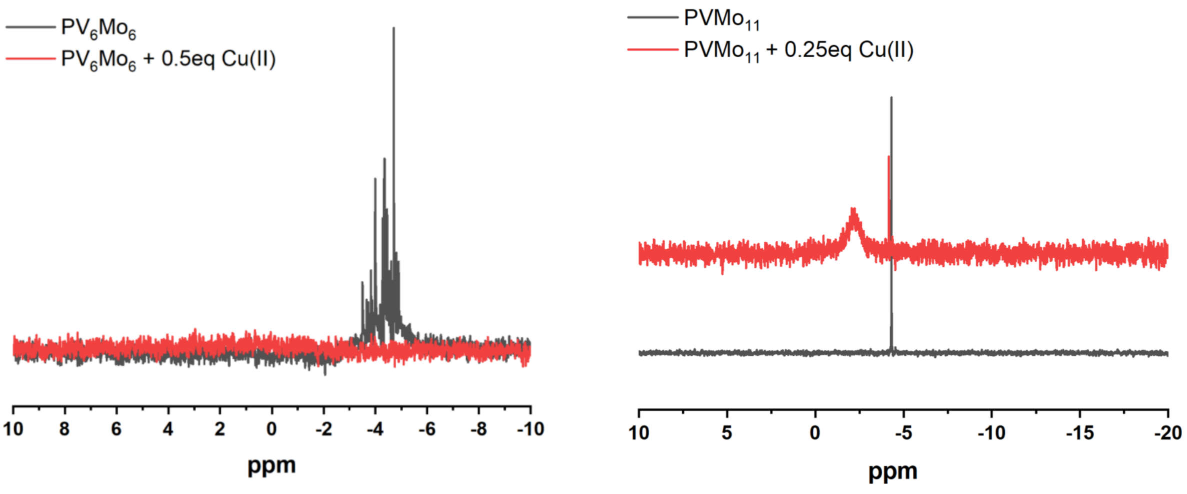

(a) (b)

Figure S23. ^{31}P NMR spectra in acetonitrile-d$_3$ with respect to 85% H_3PO_4 (0 ppm) of (a) **PV_6Mo_6** before and after adding Cu(II); (b) **$PVMo_{11}$** before and after adding Cu(II);

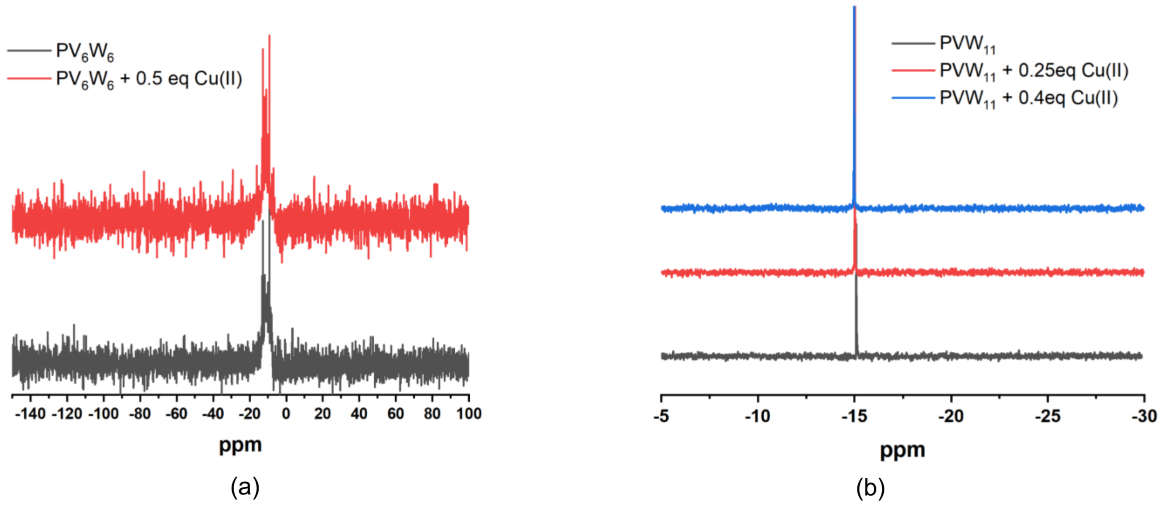

Figure S24. ^{31}P NMR spectra in acetonitrile-d$_3$ with respect to 85% H_3PO_4 (0 ppm) of (a) **PV_6W_6** before and after adding Cu(II); (b) **PVW_{11}** before and after adding Cu(II);

References

(1) Day, V. W.; Klemperer, W. G.; Maltbie, D. J. Where Are the Protons in $H_3V_{10}O_{28}^{3-}$? *J. Am. Chem. Soc.* **1987**, *109*, 2991-3002.
(2) Guo, W.; Luo, Z.; Lv, H.; Hill, C. L. Aerobic Oxidation of Formaldehyde Catalyzed by Polyvanadotungstates. *ACS Catal.* **2014**, *4*, 1151-1161.
(3) Tsigdinos, G. A.; Hallada, J. C. Molybdovanadophosphoric Acids and Their Salts. I. Investigation of Methods of Preparation and Characterization. *Inorg. Chem.* **1968**, *7* (3), 437-441.
(4) Tsigdinos, G. A. Heteropoly compounds of molybdenum and tungsten. *Top. Curr. Chem.* **1978**, *76* (Aspects Molybdenum Relat. Chem.), 1-64.
(5) Hamamoto, M.; Nakayama, K.; Nishiyama, Y.; Ishii, Y. Oxidation of Organic Substrates by Molecular Oxygen/Aldehyde/ Heteropolyoxometalate System. *J. Org. Chem.* **1993**, *58*, 6421-6425.
(6) Alston, B. Aqueous polyoxometalates: design and analysis of electrochemical catalysts for the indirect reduction of oxygen in PEM fuel cells. University of Liverpool, 2013.
(7) Yokota, T.; Fujibayashi, S.; Nishiyama, Y.; Sakaguchi, S.; Ishii, Y. Molybdovanadophosphate (NPMoV)/hydroquinone/O2 system as an efficient reoxidation system in palladium-catalyzed oxidation of alkenes. *J. Mol. Catal. A: Chem.* **1996**, *114* (1-3), 113-122.
(8) Botar, B.; Geletii, Y. V.; Kögerler, P.; Musaev, D. G.; Morokuma, K.; Weinstock, I. A.; Hill, C. L. The True Nature of the Di-iron(III) γ-Keggin Structure in Water: Catalytic Aerobic Oxidation and Chemistry of an Unsymmetrical Trimer. *J. Am. Chem. Soc.* **2006**, *128*, 11268-11277.
